# Supplementary material for: Simplifying and optimising management of acute malnutrition in children aged 6 to 59 months: study protocol for a community-based individually randomised controlled trial in Kasaï, Democratic Republic of Congo
Source: BMJ Open. 2020 Dec 2;10(12):e041213. doi: 10.1136/bmjopen-2020-041213 (PMC7713214; doi:10.1136/bmjopen-2020-041213)
Supplement: Supplementary data [file bmjopen-2020-041213supp001.pdf]

Additional file 1. Standard operating procedure for taking anthropometric measurements

OptiMA study – The Democratic Republic of Congo

	République Démocratique du Congo Ministère de la Santé; Inserm	SOP_01_OptiMA-RDC	
	Procedure for taking anthropometric measurements	Version	
		V1.0	24/05/2019

Version		Modification
1.0	24/05/2019	Creation

Objective

This document is intended to describe the process of taking anthropometric measurements in the OptiMA -DRC clinical trial.

Scope of application

These procedures are intended to be used by the research team of the OptiMA-DRC trial, and more specifically for the nurses and community health workers to follow during the study.

Acronyms

AM: Acute Malnutrition

CHW: Community Health Workers

CMAM: Community Management of Acute Malnutrition

CTT: Clinical Trial Technician

IT: Titular Nurse of health centre

MUAC: Mid Upper Arm Circumference

WHZ: Weight for Height Z score

1. General principles

From the start of the inclusion phase of the trial, research teams will organize monthly active screening for acute malnutrition (AM) in villages in the study health zones. During these sessions, the community health workers (CHWs) previously trained for this purpose will take anthropometric measurements (weight, height, mid upper arm circumference (MUAC)) and check for bilateral nutritional oedema under the supervision of a nurse among all children aged 6 to 59 months present on the screening day and accompanied by a legal representative.

Children coming for a paediatric consultation at the health centre will also be screened for acute malnutrition by health centre staff using this procedure.

Anthropometric measurements will also be taken in the children included in the study during weekly follow-up visits to the health centre and during biweekly follow-up home visits.

2. Logistics/data collection

The equipment needed to take anthropometric measurements are:

- 25 kg Salter-type scale graduated to the nearest hundredth gram;
- A standard 5 kg weight;
- Height board;
- MUAC bracelets;
- Basins for weighing and soaps for cleaning the basins;
- Registry dedicated to active screening for AM in villages as part of the OptiMA-DRC trial;
- Dedicated registry for passive AM screening in health centres as part of the OptiMA-DRC trial;
- Therapeutic follow-up sheet used for children in the study at the inclusion visit.

3. Organization of activities

Concerning active screening in the villages and home follow-up visits, the nurse will travel by motorbike to the villages in his or her health zone according to the planned tours. He will bring a Salter scale, a 5 kg weight, a height board, the register for active screening or the therapeutic sheets for home follow-up visits.

The basins will be placed in each health area by axis, according to the geographical position and distance between villages. After each daily activity, the CHWs will proceed to clean the basin.

At the level of the health centre, the health and research staff will ensure that the equipment is available and functional on a daily basis.

4. Techniques

<u>Weight:</u>

Tare the scale before weighing the 5 kg weight. Repeat the tare on the scale every 10 children weighed.

To check the accuracy of the scale:

- Put the standard 5kg weight on the scale;
- If the scale does not read '5kg', adjust the scale when empty until it gives the correct weight of 5kg.

The plastic basin attached with four cords should be close to the floor for the child to feel safe. The use of the basin is comfortable for children and more hygienic because it can be easily washed.

The weight of the child (to the nearest 100 grams) is measured using the 25 kg Salter scale attached to the basin, following these steps:

- The scale must not be exposed to the sun;

- Readjust the scale to zero before each weighing;
- Explain to the mother that her child will be weighed naked in the basin to get her real weight and ask her to take off the child's clothes;
- The mother, helped by the CHW, places the child in the basin and remains next to the basin so that the child doesn't get too agitated and to reassure him or her;
- When the child does not move anymore, read the weight to the nearest 100 g (the needle should be at eye level) and note the weight in the register.

Height:

The height will be measured to the nearest 0.1 cm with a 130 cm wooden height board with graduated mm markings on each side. It will be measured in the prone position for children under 87 cm and in the standing position for children 87 cm or taller.

Children measuring less than 87 cm (prone position, see illustration below):

- Explain the procedure to the mother and ask her to remove the child's shoes and any ornaments in the child's hair.
- Place the board flat on the floor. The child lies down in the middle of the scale with the mother's help, with the child's feet on the side of the slider.
- The assistant holds the child's head between her/his hands at ear level and holds it firmly against the fixed part of the scale, the child's hair should be compressed. The child looks straight ahead.
- The measurer places his or her hands above the child's ankles, gently stretches the legs and places one hand on the child's thighs to prevent the child from bending his or her legs.
- Holding the legs firmly, he or she pushes the slider firmly flat against the soles of the child's feet.
- To read the measurement, the slider must be perpendicular to the axis of the scale and vertical. He then reads to the nearest 0.1cm and notes in the register.

Children measuring 87 cm or more (standing position) (see illustration below):

- Explain the procedure to the mother and ask her to remove the child's shoes and any ornaments in the hair;
- Place the standing scale on a solid flat surface and if possible against a wall.
- The child stands upright in the middle of the height board and touches the vertical plane of the board.
- The assistant holds the head, shoulders, buttocks, knees, ankles against the scale while the measurer positions the head and slide. He then reads to the nearest 0.1cm and notes in the register.

Calculating the weight for height Z-score

The weight for height Z-score (WHZ) should be recorded according to the weight for height Z-score table, which is from the national CMAM protocol (2016) and was used in the OptiMA trial. The nurse refers to the weight (in kg) and height (in cm) measured to read the child's WHZ.

How to use the weight/height ratio table?

Example: a child is 63 cm tall and weighs 6.5 kg.

- Take the table, look at the first column and search for the number 63cm (=height).
- Take a ruler or sheet of paper and place it under the number 63. On this line, find the weight corresponding to the child (in this case 6.5).
- Move up the corresponding column to determine which column it is. For this example, it corresponds to the column MEDIAN WEIGHT. For this example, the child's weight is normal in relation to his or her height. Therefore, the child's weight is appropriate for his or her height.

Example: a child is 78 cm tall and weighs 8.3 kg. This child is between column -2 and -3 Z-score or between MAM and SAM. He is too thin for his height; he is <-2 (less) and >-3 (more): he has MODERATE MALNUTRITION but NOT severe malnutrition. Record the WHZ as "<-2" in the register.

NOTE: It may happen that the height is not a round number, in which case rounding should be done according to the pattern below. For example: the size 80.1 and 80.2 are rounded to 80.0; 80.3, 80.4, 80.6 and 80.7 are rounded to 80.5; 80.8 and 80.9 are rounded to 81.0.

<u>The Mid Upper Arm Circumference (MUAC):</u>

- Ask the mother to remove the clothing that covers the child's left arm. Make a mark halfway between the shoulder and the left elbow (middle of the left arm). To do this, take a piece of string (or the MUAC itself), and place one end of the string on top of (arrow 1) and the other end on the elbow (arrow 2), making sure the string is taut. Then bend the string in half, bringing the end of the elbow back towards the end of the shoulder to the point halfway between the shoulder and the elbow.
- Another method can be used. Place the 0 of the MUAC (indicated by the 2 arrows) on top of the shoulder (arrow 4) and bring the other end towards the elbow (arrow 5). Read the number on the elbow to the nearest centimetre.
- Divide this number by two to get an estimate of the midpoint between the shoulder and the elbow. Make a mark on the arm with a pen at this value (arrow 6).
- Release the child's arm and place the MUAC around the arm at the mark. Make sure that the numbers are right side up. Make sure that the MUAC touches the skin (arrow 7).
- Check the tension on the MUAC. Make sure that the tension is correct, i.e. that the MUAC is not too tight (arm compressed) or too loose (the MUAC bracelet is not touching the skin all around the arm) (arrows 7 and 8).
- Repeat each step if necessary.
- When the MUAC is correctly positioned and the tension applied is good, read and pronounce the measurement out loud to the nearest 0.1cm (arrow 10).
- Record the measurement immediately.

<u>Checking for Nutritional Oedema:</u>

Nutritional oedema is detected in the following way:

- Normal pressure is exerted with the thumb on both feet for at least three seconds.
- If the thumb impression persists on both feet after removing the thumbs, then the child has oedema.
- Only children with bilateral oedema are recorded as having oedema.

You have to test with the pressure of your thumb! It is not enough just to look!

Severity of Oedema	Coding
**Mild Oedema**: present in both feet	**+**
**Moderate Oedema**: present in both feet and the lower part of both legs, and both hands and the lower part of both forearms. Intermediate between mild and severe oedema ++	**++**
**Severe Oedema:** generalized, including both feet, legs, hands, arms and face	**+++**
