# Supplementary material for: Simplifying and optimising management of acute malnutrition in children aged 6 to 59 months: study protocol for a community-based individually randomised controlled trial in Kasaï, Democratic Republic of Congo
Source: BMJ Open. 2020 Dec 2;10(12):e041213. doi: 10.1136/bmjopen-2020-041213 (PMC7713214; doi:10.1136/bmjopen-2020-041213)
Supplement: Supplementary data [file bmjopen-2020-041213supp002.pdf]

Additional file 2: RUTF ration per week according to Standard and OptiMA strategy

Standard RUTF ration per week		OptiMA RUTF ration per week			
	MUAC <115 or oedema or WHZ < -3 Z score		MUAC < 115 mm or oedema	MUAC [115-119 mm]	MUAC >=120 mm
**Weight (KG)**	**Number of RUTF sachet per week**	**Weight (KG)**	**Number of RUTF sachet per week**		
**3.0-3.4**	9	**3.0-3.4**	10	8	7
**3.5 - 4.9**	11	**3.5 - 4.0**	11	8	7
		**4.1 - 4.4**	12	9	7
		**4.5 - 4.9**	13	10	7
**5.0 - 6.9**	14	**5.0 - 5.4**	14	11	7
		**5.5 - 5.9**	15	12	7
		**6.0 - 6.4**	16	12	7
		**6.5 - 6.9**	17	13	7
**7.0 - 9.9**	21	**7.0 -7.4**	18	13	8
		**7.5 - 7.9**	19	14	8
		**8.0 - 8.4**	20	15	9
		**8.5 - 8.9**	22	15	9
		**9.0 - 9.4**	23	16	9
		**9.5 - 9.9**	24	17	9
**10.0 - 14.9**	28	**10.0 - 10.4**	25	18	10
		**10.5 - 10.9**	26	19	10
		**11.0 -11.4**	27	20	10
		**11.5 - 11.9**	29	21	10
		**12.0 - 12.4**	30	22	11
		**12.5 - 12.9**	31	22	11
		**13.0 - 13.4**	33	23	12
		**13.5 - 13.9**	34	24	12
		**14.0 - 14.4**	35	25	12
		**14.5 -14.9**	36	26	13
**15.0 - 19.9**	35	**15.0 - 15.4**	36	28	14
		**15.5 - 15.9**	36	28	14
		**16.0 - 16.4**	36	28	14
		**16.5 - 16.9**	36	28	14
		**17.0 - 17.4**	36	28	14
		**17.5 - 17.9**	36	28	14
		**18.0 - 18.4**	36	28	14
		**18.5 - 18.9**	36	28	14
		**19.0 - 19.4**	36	28	14
		**19.5 - 19.9**	36	28	14
