# Supplementary material for: Simplifying and optimising management of acute malnutrition in children aged 6 to 59 months: study protocol for a community-based individually randomised controlled trial in Kasaï, Democratic Republic of Congo
Source: BMJ Open. 2020 Dec 2;10(12):e041213. doi: 10.1136/bmjopen-2020-041213 (PMC7713214; doi:10.1136/bmjopen-2020-041213)
Supplement: Author's manuscript [file bmjopen-2020-041213.draft_revisions.pdf]

BMJ Open is committed to open peer review. As part of this commitment we make the peer review history of every article we publish publicly available.

When an article is published we post the peer reviewers' comments and the authors' responses online. We also post the versions of the paper that were used during peer review. These are the versions that the peer review comments apply to.

The versions of the paper that follow are the versions that were submitted during the peer review process. They are not the versions of record or the final published versions. They should not be cited or distributed as the published version of this manuscript.

BMJ Open is an open access journal and the full, final, typeset and author-corrected version of record of the manuscript is available on our site with no access controls, subscription charges or pay-per-view fees (http://bmjopen.bmj.com).

If you have any questions on BMJ Open's open peer review process please email info.bmjopen@bmj.com

BMJ Open

Simplifying and optimizing management of acute malnutrition in children aged 6 to 59 months: study protocol for a community-based individually randomized-controlled trial in Kasaï, Democratic Republic of Congo

Journal:	*BMJ Open*
Manuscript ID	bmjopen-2020-041213
Article Type:	Protocol
Date Submitted by the Author:	03-Jun-2020
Complete List of Authors:	CAZES, Cécile; University of Bordeaux College of Health Sciences, Inserm, French National Research Institute for Sustainable Development (IRD), Bordeaux Population Health Research Center, Team IDLIC, UMR 1219<br>PHELAN, Kevin; ALIMA, Alliance for International Medical Action<br>Hubert, Victoire; ALIMA, Alliance for International Medical Action<br>ALITANOU, Rodrigue; ALIMA, Alliance for International Medical Action<br>BOUBACAR, Harouna; ALIMA, Alliance for International Medical Action<br>IZIE BOZAMA, Liévin; Ministry of Health, National Nutrition Programme (PRONANUT)<br>TSHIBANGU SAKUBU, Gilbert; Kamuesha health zone in the Kasaï province, Ministry of Health<br>BEUSCART, Aurélie; University of Bordeaux College of Health Sciences, Inserm, French National Research Institute for Sustainable Development (IRD), Bordeaux Population Health Research Center, Team IDLIC, UMR 1219<br>YAO, Cyrille; University Hospital Center of Treichville, PACCI research programme<br>Gabillard, Delphine; University of Bordeaux College of Health Sciences, Inserm, French National Research Institute for Sustainable Development (IRD), Bordeaux Population Health Research Center, Team IDLIC, UMR 1219<br>KINDA, Moumouni; ALIMA, Alliance for International Medical Action<br>AUGIER, Augustin; ALIMA, Alliance for International Medical Action<br>ANGLARET, Xavier; University of Bordeaux College of Health Sciences, Inserm, French National Research Institute for Sustainable Development (IRD), Bordeaux Population Health Research Center, Team IDLIC, UMR 1219<br>Shepherd, Susan; ALIMA, Alliance for International Medical Action<br>Becquet, Renaud; University of Bordeaux College of Health Sciences, Inserm, French National Research Institute for Sustainable Development (IRD), Bordeaux Population Health Research Center, Team IDLIC, UMR 1219
Keywords:	Nutrition < TROPICAL MEDICINE, PUBLIC HEALTH, Clinical trials < THERAPEUTICS, Paediatric pathology < PAEDIATRICS, Community child health < PAEDIATRICS, Protocols & guidelines < HEALTH SERVICES ADMINISTRATION & MANAGEMENT

SCHOLARONE™
Manuscripts

I, the Submitting Author has the right to grant and does grant on behalf of all authors of the Work (as defined in the below author licence), an exclusive licence and/or a non-exclusive licence for contributions from authors who are: i) UK Crown employees; ii) where BMJ has agreed a CC-BY licence shall apply, and/or iii) in accordance with the terms applicable for US Federal Government officers or employees acting as part of their official duties; on a worldwide, perpetual, irrevocable, royalty-free basis to BMJ Publishing Group Ltd ("BMJ") its licensees and where the relevant Journal is co-owned by BMJ to the co-owners of the Journal, to publish the Work in this journal and any other BMJ products and to exploit all rights, as set out in our licence.

The Submitting Author accepts and understands that any supply made under these terms is made by BMJ to the Submitting Author unless you are acting as an employee on behalf of your employer or a postgraduate student of an affiliated institution which is paying any applicable article publishing charge ("APC") for Open Access articles. Where the Submitting Author wishes to make the Work available on an Open Access basis (and intends to pay the relevant APC), the terms of reuse of such Open Access shall be governed by a Creative Commons licence – details of these licences and which Creative Commons licence will apply to this Work are set out in our licence referred to above.

Other than as permitted in any relevant BMJ Author's Self Archiving Policies, I confirm this Work has not been accepted for publication elsewhere, is not being considered for publication elsewhere and does not duplicate material already published. I confirm all authors consent to publication of this Work and authorise the granting of this licence.

For peer review only

Simplifying and optimizing management of acute malnutrition in children aged 6 to 59 months: study protocol for a community-based individually randomized-controlled trial in Kasaï, Democratic Republic of Congo

Running title: OptiMA-DRC

Cécile CAZES [1], Kevin PHELAN [2], Victoire HUBERT [3], Rodrigue ALITANOU [3], Harouna BOUBACAR [3], Liévin IZIE BOZAMA [4], Gilbert TSHIBANGU SAKUBU [5], Aurélie BEUSCART [1], Cyrille YAO [6], Delphine GABILLARD [1], Moumouni KINDA [7], Augustin AUGIER [2], Xavier ANGLARET [1], Susan SHEPHERD [7], Renaud BECQUET [1]

1. University of Bordeaux, Inserm, French National Research Institute for Sustainable Development (IRD), Bordeaux Population Health Research Center, Team IDLIC, UMR 1219, Bordeaux, France
2. Alliance for International Medical Action (ALIMA), Paris, France
3. Alliance for International Medical Action (ALIMA), Kamuesha, Democratic Republic of Congo
4. National Nutrition Programme (PRONANUT), Ministry of Health, Kinshasa, Democratic Republic of Congo
5. Kamuesha health zone in the Kasaï province, Ministry of Health, Kamuesha, Democratic Republic of Congo
6. PACCI research programme, University Hospital of Treichville, Abidjan, Ivory Coast
7. Alliance for International Medical Action (ALIMA), Dakar, Senegal

Address for correspondence:

Cécile CAZES, MPH

Bordeaux Population Health Research Center – Team IDLIC

University of Bordeaux

146 rue Léo Saignat,

33000, Bordeaux, France

Email: cecile.cazes@u-bordeaux.fr

Contact and address of the trial sponsor:

Plateforme CORAL - Clinical & Operational Research Alliance

ALIMA - The Alliance for International Medical Action, Dakar, Sénégal et

Inserm- U1219 Bordeaux Population Health, Université de Bordeaux, France

15 rue des immeubles industriels 75011 Paris

Email : claire.levy-marchal@coral.alima.ngo

For peer review only - http://bmjopen.bmj.com/site/about/guidelines.xhtml

Word count: 3996 words (from background to discussion)

Abstract

Introduction

Acute malnutrition (AM) affects an estimated 50 million children under five years of age worldwide but only about 20% receive treatment. AM is a continuum condition, arbitrarily divided into moderate and severe (SAM) categories, funded and managed in separate programs under different protocols. Optimizing acute MAlnutrition treatment (OptiMA) aims to simplify and optimize AM management by treating children with mid-upper arm circumference (MUAC) <125 mm or edema with one product – ready-to-use therapeutic food– at a gradually tapered dose. Our main objective is to compare the OptiMA strategy with the standard nutritional protocol in children 6-59 months with MUAC <125 mm or oedema without additional complications, as well as in children classified as uncomplicated SAM (i.e. MUAC <115 mm or WHZ <-3 Z-score or with oedema).

Methods and Analysis

This study is a non-inferiority individually randomized-controlled clinical trial conducted at community level in the Democratic Republic of Congo. Children 6-59 months with MUAC < 125 mm or WHZ <-3 Z-score or with bipedal oedema and without medical complication are included after signed informed consent in outpatient health facilities after screening in their village or during outpatient paediatric visits. All participants are followed for 6 months. For the primary objective, 890 participants are needed, with 480 SAM children needed for the main secondary objective. Success in both arms is defined at 6 months post inclusion as being alive, not acutely malnourished per the definition applied at inclusion, and without additional episode of acute malnutrition throughout the 6-month observation period. Recovery is the main secondary outcome. We will realise non-inferiority analyses in per-protocol and intention-to-treat for both outcomes.

Ethics and dissemination

Ethics approvals was obtained for this study protocol. We will submit results for publication in peer-reviewed journal and disseminate findings in international and national conferences and meetings.

Trial registration: NCT03751475. Registered 19 September 2018, https://clinicaltrials.gov/ct2/show/NCT03751475

Keywords: Acute malnutrition, child, MUAC, RUTF, Africa, clinical trial, protocol

For peer review only - http://bmjopen.bmj.com/site/about/guidelines.xhtml

Strengths and limitations of this study

- This is the first study of a MUAC-based malnutrition treatment protocol with gradually tapered dose of RUTF that uses an individually randomized control design.
- The main outcome takes into account sustained health and nutrition status at 6 months after inclusion, including post-recovery relapse, and spontaneous recovery for children who are not eligible for RUTF supplementation.
- The methodology used for randomization (i.e. stratification based on WHO definition of SAM) allows for two major analyses with sufficient robustness: first how all children with MUAC <125 mm or WHZ <-3 or oedema respond under OptiMA and second how children only meeting the WHO definition of SAM respond.
- This trial is not a multisite study and only takes place in one country, the Democratic Republic of Congo.

Background

Acute malnutrition (AM) affects an estimated 50 million children under five years of age, including 16.6 million severe cases, and is an underlying cause of 800,000 deaths each year worldwide (1,2). Other estimation methods based on incidence instead of prevalence suggest the caseload for this major public health concern may be substantially higher (3,4). One quarter of all acutely malnourished children are in Africa and as many as 2 million severely wasted children were reported in the Democratic Republic of Congo (DRC) in 2018 (5).

Since the United Nations (UN) Joint Statement in 2009, millions of children have received treatment for severe acute malnutrition (SAM) via standard community-based management (6,7) with >80% of children recovering in outpatient care (8). Despite more than a decade of expanded access to treatment, coverage of nutrition programs remains remarkably low in a general context of chronic underfunding. As few as 20% of wasted children actually receive nutritional treatment (9,10) and the World Bank estimates that it would take an additional US$6.3 billion per year to reach a coverage of 80% (11). There is therefore an urgent need to better allocate available resources through the simplification and optimization of current acute malnutrition protocols. Adapting programming along three aspects of case-management may allow for further improved access to treatment for acutely malnourished children.

For peer review only - http://bmjopen.bmj.com/site/about/guidelines.xhtml

First, acute malnutrition is as a continuum condition, but it is arbitrarily divided into moderate (MAM) and severe (SAM) categories. Such separation results in separate MAM and SAM programs overseen by different UN agencies and using different protocols and products, ready to use supplementary food (RUSF) or fortified-blended flours, and ready to use therapeutic food (RUTF) for MAM and SAM children, respectively. This complicates supply chain, delivery of care, data collection and creates confusion and supplementary workload for caregivers and health workers alike (12). In practice, treatment is often available for SAM children only, resulting in lost opportunities for saving lives whereas an earlier identification and treatment of children in the wasting process would have led to fewer hospitalizations and deaths. The treatment of MAM was indeed shown to be cost-effective in reducing mortality risk by more than 10% (13).

Second, the current case definitions are un-necessarily complicated. Confusion is further exacerbated by a complicated case definition to determine program eligibility, using two independent anthropometric criteria: the middle upper arm circumference (MUAC) or the weight-for-height Z-score (WHZ). WHZ alone or in combination with MUAC does not offer a clear advantage for identifying children at near-term risk of death over MUAC alone (14). MUAC-only programming is expanding as evidence accumulates that weight gain and MUAC gain track each other and respond to treatment in similar ways (15–17). MUAC is therefore becoming a stand-alone practical tool for all phases of nutrition programming: screening, admission, monitoring recovery and determining discharge. Furthermore, simplicity of the use of MUAC bracelets makes it possible to screen children at community level, even at home, by previously trained mothers (18).

Third, therapeutic food rations could be allocated more efficiently. The RUTF ration for treating SAM (130-200 kcal/kg/day) is paradoxical. The amount of RUTF prescribed remains constant or even increases with weight gain up until discharge, i.e. as the child recovers from acute malnutrition, while the trajectory of weight and MUAC gain is maximal during the first 2-3 weeks of therapeutic supplementation and the average weight gain then decreases (19). Consequently, gradual dose reduction seems to be a more rational use of RUTF. This would allow an efficiency gain as less severe children would receive smaller rations of RUTF, which accounts for a large proportion of total malnutrition programme costs (20).

Results on integrating SAM and MAM treatment in a single MUAC-based protocol using RUTF only with an optimized dosage were firstly published in peer-review journal in 2015. This cluster-randomized trial conducted in Sierra Leone found high recovery rates, a reduced caseload of SAM and MAM, a better coverage rate and a reduction of RUTF costs when compared to the standard protocol (21). More recently,

For peer review only - http://bmjopen.bmj.com/site/about/guidelines.xhtml

a non-inferiority randomized trial in Burkina Faso showed that the reduction of the RUTF dose after the first two weeks results in similar weight gain velocity and recovery rates as with the standard dose given throughout SAM treatment (22).

Our research consortium developed the Optimizing treatment for Acute Malnutrition (OptiMA) strategy that simplifies the definition of acute malnutrition to a MUAC < 125mm or the presence of bipedal oedema, and uses one product only for treatment (RUTF) at a gradually reduced dose based on child's weight and MUAC status **(see Table 1).** The OptiMA strategy was first implemented in a proof-of-concept single-arm trial at district level in Burkina-Faso and showed a recovery rate that exceeded national Burkina Faso and international SPHERE standards for both SAM and MAM (86.3%; 95%CI: 85.4-87.2) with excellent health worker compliance to the RUTF dosage table specifically designed for this strategy (23). However, recovery of children admitted at MUAC<115mm was poorer than anticipated (70.4%; 95 % CI 67.5-73.5) and there is now a need for a randomized controlled trial to compare the OptiMA strategy to current national malnutrition protocols. We therefore developed this "OptiMA-DRC" non-inferiority individually randomized clinical trial protocol to compare the OptiMA strategy to the DRC nutritional standard protocol in uncomplicated wasted 6-59-month-old children.

We hypothesized that the OptiMA strategy would be as effective as the DRC standard protocol currently in use, in children 6 to 59 years old as judged by the success rate in the treatment of uncomplicated severe and moderate acute malnutrition at 6 months post-randomization and in terms of recovery rate in the treatment of uncomplicated severe acute malnutrition.

Table 1: Wasting definition, treatment products, calculation of dosage and recovery definition in the DRC national and OptiMA protocol

	National DRC Protocol		OptiMA Protocol		
	**SAM**	**MAM**	**Acute malnutrition**		
**Wasting definition**	MUAC<115mm<br>Or WHZ<-3 (SD)<br>Or Bipedal oedema	MUAC [115mm-124]<br>Or -3 < WHZ <-2	MUAC < 125mm<br>Or Bipedal oedema		
**Treatment product**	RUTF<br>150-200 Kcal/kg/d	Super cereal plus<br>200 g/d<br>(~1000Kcal/d)<br>Or	MUAC < 115 mm<br>Or bipedal oedema	MUAC [115-119 mm]	MUAC [120-124 mm]

For peer review only - http://bmjopen.bmj.com/site/about/guidelines.xhtml

		RUSF, one 92g sachet /d (500Kacl/d)	RUTF 170-200 (Kcal/Kg/d)	RUTF 125-190 (Kcal/Kg/d)	RUTF 50-166 (Kcal/Kg/d)
**Calculation of dosage**	According to the weight	Fixed amount, regardless of weight or MUAC status	According to MUAC status and weight		
**Recovery definition**	MUAC≥125mm Or WHZ ≥-1.5 Z score	MUAC≥125mm Or WHZ ≥-1.5 Z score If After recovery from SAM: MUAC≥125mm And WHZ ≥-1.5 Z score And discharge after 3 months.	MUAC≥125mm for two consecutive weeks		
	No oedema for two consecutive weeks and Minimum 4 weeks in program and good clinical health		No oedema for two consecutive weeks and Minimum 4 weeks in program and good clinical health		

Objectives

The principal objective aims to determine, 6 months after inclusion, whether the OptiMA strategy, which adapts RUTF dosage according to MUAC and weight in wasted and uncomplicated children 6-59 months of age with bipedal oedema or a MUAC < 125 mm, provides a success rate that is non-inferior to that of the standard DRC protocol in use in the same outpatient health facilities. The definition of success is provided in the Study Outcomes section.

The main secondary objective is to determine whether the recovery rate of uncomplicated severely wasted children according to the current World Health Organization (WHO) definition (24) (MUAC <115 mm or WHZ<-3 standard deviation (SD) Z-score or bilateral oedema) managed according to the OptiMA strategy is not inferior to that of the national standard protocol.

Other secondary objectives aim to describe and to compare between both protocols RUTF consumption, relapses occurring after recovery, international SPHERE standards outcomes, recovery and success in

For peer review only - http://bmjopen.bmj.com/site/about/guidelines.xhtml

children combining wasting and stunting at inclusion, and to describe the nutritional and clinical status of children hospitalized during follow-up.

Methods

This protocol follows the Standard Protocol Items: recommendations for Interventional Trials (SPIRIT) guidelines (25).

Design

This study is a non-inferiority individually randomized-controlled clinical trial conducted at health centre and community level. Participants are randomly assigned to either the OptiMA strategy arm (intervention = OptiMA) or the national standard protocol arm (control = Standard).

Study Setting

The trial is located in the Kamuesha health zone, Kasai province in DRC and nested within a medical and nutritional emergency humanitarian project launched by ALIMA in May 2018. ALIMA supports the DRC Ministry of Health (MoH) in 9/26 health areas in the Kamuesha health zone to address a nutritional crisis occurring after a two-year-long armed conflict and important population displacements (26). During trial preparation phase, we visited all the health areas covered by ALIMA and selected four as research sites based on the following criteria. We considered demographic, epidemiological and logistical data and observations provided by local stakeholders in order to facilitate compliance and monitoring of this protocol. According to projections from the last census, the four health areas chosen covered a population of 65,000 people, including 12,000 children aged 6 to 59 months, spread over 60 villages. Health facilities included in the study were the four community-based primary health centres of these four health areas alongside with the general hospital of the district of Kamuesha for referring participants with medical complications. Most (N=49) villages were less than 10 kilometres away from the primary health centres while some (N=11) were more than 15 kilometres away. The Kamuesha health zone is located in a tropical forest and is particularly isolated.

Eligibility criteria

Children aged 6–59 months, residing in one of the four health areas and meeting at least one of the three following acute malnutrition criteria were eligible for inclusion in the trial: MUAC <125 mm and/or Bilateral pitting oedema (+/++) and/or WHZ<-3SD. Children with a medical complication requiring hospitalization, negative appetite test, oedema grade +++, with a known allergy to milk or peanuts or RUTFs, with a known chronic pathology (such as sickle cell anaemia, trisomy 21, congenital heart disease, neurological disease),

For peer review only - http://bmjopen.bmj.com/site/about/guidelines.xhtml

or already enrolled in a malnutrition programme were excluded from the trial. Two categories of children were included in the trial but not eligible for randomization for ethical concerns. First, children with both WHZ<-3SD and MUAC ≥125mm but no oedema were systematically included in the nutritional standard protocol arm, as they were not RUTF eligible according to the wasting definition of the OptiMA strategy. Second, children with a sibling already included in the study were systematically allocated to the study arm of the index sibling previously included in the trial.

Study outcomes

The primary outcome is judged by a binary composite indicator. Children classified as 'success' fulfil all of the following criteria: alive, not acutely malnourished per the definition applied at inclusion and no additional episode of acute malnutrition throughout the 6-month observation period. All other children are classified unsuccessful.

The main secondary outcome will be determined among children in both arms of the trial who fulfil the current WHO definition of SAM. For this sub-group, recovery is defined after a 4-week minimum duration of treatment as clinically well, absence of bipedal oedema and for the OptiMA arm a MUAC ≥ 125 mm or for the standard arm MUAC ≥125 mm or WHZ ≥-1.5Z.

Other secondary outcomes are cost-efficiency indicators linked to RUTF as: mean consumption of RUTF per children in success and per SAM children recovered and average number of SAM children cured for an equivalent amount of RUTF in both strategies. We will also look at relapse rate throughout the 3-month period following the first recovered SAM episode, average weight and MUAC gain, average daily MUAC gain in both strategies, recovery and success rates in children facing both stunting and wasting at inclusion **(see Table 2)**.

Table 2: Outcomes

	Measurement variable	Denominator	Method of aggregation	Time point
**Primary**				
Success	Composite : alive, not acutely malnourished per the definition applied at inclusion and no additional episode of acute malnutrition throughout the 6- month observation period	Children with MUAC < 125 or WHZ <-3 Z score or bipedal oedema at inclusion	Proportion	6 months post-inclusion
**Main secondary**				

For peer review only - http://bmjopen.bmj.com/site/about/guidelines.xhtml

For peer review only

Recovery	Composite: a 4 week minimum duration of RUTF treatment, absence of bipedal oedema and for the OptiMA arm a MUAC $\geq$ 125 mm and for the standard arm MUAC $\geq$ 125 mm or WHZ $\geq$-1.5 Z score	Children with MUAC < 115 or WHZ <-3 Z score or bipedal oedema at inclusion	Proporti on	End of RUTF treatment initiated at inclusion
**Secondary**				
Consumption of RUTF per children in success	Sachets of RUTF consumed	Children with MUAC < 125 or WHZ <-3 Z score or bipedal oedema at inclusion	Mean	6 month post-inclusion
Consumption of RUTF per SAM children at inclusion recovered	Sachets of RUTF consumed	Children with MUAC < 115 or WHZ <-3 Z score or bipedal oedema at inclusion	Mean	End of RUTF treatment initiated at inclusion
Average number of children in success for an equivalent amount of RUTF	Children cured with 1500 sachets of RUTF	Children with MUAC < 125 or WHZ <-3 Z score or bipedal oedema at inclusion	Mean	6 month post-inclusion
Average number of MAS children at inclusion cured for an equivalent amount of RUTF	Children cured with 1500 sachets of RUTF	Children with MUAC < 115 or WHZ <-3 Z score or bipedal oedema at inclusion	Mean	End of RUTF treatment initiated at inclusion
Relapse to SAM WHO definition	Children with MUAC<115 or WHZ <-3 Z score or oedema after RUTF treatment	Children with MUAC < 115 or WHZ <-3 Z score or bipedal oedema at inclusion	Proporti on	After a 3 months period following the cured SAM episode at inclusion
Average daily weight gain in SAM children recovered	g/kg/day	Children with MUAC < 115 or WHZ <-3 Z score or bipedal oedema at inclusion	Daily	End of RUTF treatment initiated at inclusion
Average daily MUAC gain in SAM children recovered	mm/day	Children with MUAC < 115 or WHZ <-3 Z score or	Daily	End of RUTF treatment

For peer review only - http://bmjopen.bmj.com/site/about/guidelines.xhtml

	bipedal oedema at inclusion	initiated at inclusion

Sample size considerations

We determined the sample size for the principal objective using an expected success rate of 55% and for the secondary main objective an expected recovery rate of 85%. Both of these hypotheses are based on recovery, death, default and relapse rates available in the literature for severe and moderately wasted children supplemented with RUTF and/or RUSF in DRC and other African contexts (27,28). We also considered spontaneous recovery rate documented for MAM children without treatment in the standard arm (29). Indeed, during the preparation phase for this trial, we visited twice the four sites included in the study and found that standard program for MAM was not or partially functional.

Based on both statistical reasoning and clinical judgement, we assumed a margin of non-inferiority of 10%, the same as the three other non-inferiority trials assessing a new simplified strategy with RUTF in wasted children (30–32).

To demonstrate non-inferiority of the OptiMA strategy compared to standard protocol, 890 and 490 participants are required for the principal and main secondary objectives, respectively, with 80% power at a unilateral significance level of 2,5% and with an inflation of 15% to account for un-exploitable data. We estimated 15% of incomplete data in the light of the tough reality of this study setting where the population faced volatile security, internal displacement and geographical barriers to access health facilities.

Randomization

The trial statistician established and kept the randomization list confidential. Randomization was done in blocks, and was stratified by the severity of acute malnutrition according to the WHO definition on the study site. This double stratification will allow for recruitment of comparable groups of SAM and non-SAM children and for simultaneous randomization in the four sites. The trial data scientist integrated the list into a randomization software developed for the trial that was set up in four tablet devices. This software assigned a treatment arm by sequentially drawing from this list each time a randomization procedure was completed. Once the sample size for the primary objective was reached, only SAM cases continued to be enrolled and randomized until the sample size required for the main secondary objective was attained.

Patient and Public Involvement

To ensure the trial is understood, accepted and in accordance with the local context, meetings on the methodology were organized with community representatives in each village and with nutrition actors prior

For peer review only

For peer review only - http://bmjopen.bmj.com/site/about/guidelines.xhtml

to recruitment. During active phase, regular oral presentation on progress of the trial are held at local level. We intend to disseminate the findings through meetings with the community representative and national nutrition actors.

Enrolment and informed consent procedure

Prior to enrolment, all community health workers nurse research officers and MoH medical staff implicated in the study were trained on the trial protocol, standardized operating procedures and the correct use of tools developed for data collection. Nurse research officers were specifically, trained to enrol, randomize study participants, follow home and outpatient visits. Children were recruited through active mass malnutrition screening organized monthly in each of the 60 villages. Concomitantly, children attending outpatient consultation in each of the four study health centres underwent routine passive malnutrition screening and were also eligible for recruitment in the trial. In both active and passive screening, a nurse research officer, helped by a community health worker, collected anthropometric (weight, height, MUAC, WHZ) and clinical (presence of oedema) information on children aged 6 to 59 months who then underwent a medical consultation to check for their eligibility in participating in the trial. A standard operating procedure **(see additional files 1)** for assessing child anthropometry (weight, height, MUAC and oedema) was implemented in each site. For each eligible child, the caretaker was given a study information sheet detailing the aims, treatment, study duration and frequency of clinic and village follow-up. This information was explained orally in Tshiluba (local language). Caretakers who agreed to participate in the study indicated their consent by signing (signature or fingerprint) a written consent form. When caretakers were not able to read or write, an impartial witness oversaw the consent process and attested to the caretaker's consent by signing the consent form on her/his behalf. All caretakers were informed about their right to withdraw from the study at any time without affecting the quality of medical care delivered to their children. All medical care and nutritional treatment are provided free of charge to the family, regardless of participation in the study.

Intervention and follow-up

In the intervention arm, all children with a MUAC<125mm or oedema are supplemented with RUTF, according to the OptiMA dosage table based on the progression of MUAC and weight gain during recovery (RUTF dosage is gradually reduced as weight and MUAC increased) **(see Table 1).** OptiMA RUTF dosage table consists in giving a daily caloric intake of 170-200, 125-190 and of 50-166 Kcal/Kg/day in children with a MUAC <115 mm, between 115 and 119 mm and >119mm, respectively. Children with oedema receive the same RUTF ration as children with MUAC < 115 mm until oedema resolved, at which point their ration is determined by MUAC and weight **(see additional files 2).**

For peer review only - http://bmjopen.bmj.com/site/about/guidelines.xhtml

In the control arm, children included with MUAC<115 or WHZ<-3SD or nutritional oedema, are treated with RUTF, according to the standard dosage table based on weight at each visit (RUTF dosage gradually increased as weight increased). DRC protocol for acute malnutrition treatment provides a RUTF daily caloric intake of 150-200 Kcal/kg/day during all treatment duration until recovery in severely wasted children and one sachet of RUSF per day (500Kcal/day) in moderately wasted children.

The differences between the OptiMA and the DRC national standard protocols, summarized in Table 1, concern treatment eligibility and definitions of recovery, the type of therapeutic food and RUTF dosage calculation. Apart from these, the standard nutrition protocol is applied to all children in both arms. All severely wasted cases (WHO definition) receive Amoxicillin 50-100 mg/kg/day for 7 days, and all children receive vitamin A and deworming. In both arms, a malaria rapid testing is systematically performed at inclusion and at any point during follow-up in case clinical signs of malaria occur; if positive, an Artemisinin-combination treatment is administrated to the child.

All participants are followed for 6 months after their inclusion **(see Figure 1).** Children receiving nutritional treatment come weekly (or every two weeks for those living in villages more than 14 km away from the health centre) for an outpatient consultation until the end of the nutritional treatment. After this facility-based follow-up, or immediately after inclusion for children with moderate acute malnutrition who do not receive nutritional treatment in the standard arm, children are consulted every two weeks in their villages through study completion. At village level, a nurse research officer assisted by one or two community health workers monitor anthropometric and clinical status of these children and refer to the primary health facility or to the Kamuesha general hospital those who need appropriate nutritional and medical care.

Blinding

This trial is fully unblinded. Caretakers know the RUTF dosage they give to their children, according to the intervention or standard protocols. Upon inclusion, the nurse research officer gives a trial participant card to the caretaker that specifies the nutritional strategy followed by the participant. Thanks to this card, in case of a spontaneous outpatient or inpatient consultation during follow-up in the study, nurses or doctors at any health facility are able to identify the nutritional treatment followed by the child enrolled in the study.

Data collection and management

After obtaining individual informed consent, all socio-demographic characteristics, inclusion criteria, anthropometric, clinical data collected during follow up are documented in the case report forms by the trained research officers and the trained heath providers. All data entered into the database are securely

For peer review only - http://bmjopen.bmj.com/site/about/guidelines.xhtml

transferred and stored on a secure server hosted by the methodology and management centre located in Abidjan, Côte d'Ivoire. Data and adverse events, including deaths and hospitalisations, are rigorously monitored on-site and remote according to the data monitoring plan. To ensure patient safety and data integrity, the methodology and coordinating centre continuously supervises data management activities and both senior scientific project leader and investigators performed field visits on a regular basis. If children fail to attend follow-up visits, the health community worker living in the village visits the household to ask about the reasons for absence and to encourage the caretakers to go for a consultation to check the nutritional and the clinical status of their children according to the follow-up calendar.

Statistical analyses

All statistical analyses will be performed using RStudio software (Inc., Boston, Massachusetts, USA) and per the data analysis plan established prior to performing any analysis. The number and proportion of available data will be described for each variable. Statistical tests will be carried out bilaterally with an alpha risk of 5%, and unilaterally with an alpha risk of 2, 5% for the non-inferiority analysis. Qualitative variables will be described in terms of numbers, percentages, and provided with confidence intervals when relevant. If necessary, comparisons of qualitative variables will be made using tests of chi-2, chi-2 corrected or exact Fisher test. Quantitative variables will be described in terms of mean, standard deviation and confidence interval or median, range and interquartile range. If necessary, comparisons of quantitative variables will be made using the Student's test, the Wilcoxon's test or the Kruskal-Wallis test, depending on the distribution of the variable of interest. Time delay type variables will be described in terms of the incidence of occurrence, and the probability of occurrence over time, estimated by the Kaplan-Meier method. If necessary, probability comparisons will be made using log-rank tests, or proportional risk models, after verification of the assumption of proportionality of risks.

Primary analysis of the primary and secondary main outcomes

Descriptive summaries of participant characteristics by arm at inclusion will be tabulated. Participant included in the main analyses will be described according to the diagram defined by the CONSORT recommendations (33). The occurrence of the primary and the secondary main outcome "success" and "recovery" will be compared between the two randomization arms. These two comparisons will be performed on an "intention to treat" basis (including all randomized participants), and on a "per-protocol" basis (including only those who received the full randomized treatment strategy). Non-inferiority analyses will be performed. If non-inferiority is demonstrated, the other secondary analyses will be performed, if

For peer review only - http://bmjopen.bmj.com/site/about/guidelines.xhtml

appropriate, as a superiority analysis. Superiority will be particularly sought for cost outcomes related to RUTF cost-efficiency.

Sensitivity analysis of the primary outcome

Success and recovery will be analysed based on all available data first. A sensitivity analysis will be performed using the maximum bias method. In this analysis, deceased participants, those who have withdrawn their consent, who have been transferred to another health facility or have been lost to follow-up, will be considered to have systematically failed to the allocated treatment.

Safety

The trial is monitored monthly by an international steering committee and quarterly by a national steering committee and adverse effects are presented. No interim analysis is planned before the final analyses. The DSMB may request an interim analysis if it deems it necessary for patient safety.

The protocol states an early stopping rule of the intervention treatment in both cases: if children with SAM criteria at inclusion presents a second episode of weight loss > 5% between 2 visits and after hospitalisation and if children with no SAM criteria at inclusion reach them during RUTF supplementation.

Ethics and dissemination

We obtained ethical approval and its annual renewal from DRC national ethics committee (CNES) and from the Ethics Evaluation Committee of the French National Institute for Health and Medical Research (INSERM). In November 2019, the CNES performed an audit at each site. All data are anonymized when entered into the database using unique identification numbers. We intend to disseminate the results regardless of positive or negative findings broadly via peer-reviewed publication, conferences, and clinical networks targeting academics, policy- makers, clinicians and caregivers.

Discussion

This trial is grounded in three foundational principles: a single program for acute malnutrition using one anthropometric admission criterion and one type of therapeutic food. This study will add to evidence already available from similar studies published or in progress in Sierra Leone, Burkina Faso, South Sudan and Kenya (30,34) . Given that the profile of acute malnutrition is highly contextual, it is important to conduct similar trials in multiple settings. This will be the first such trial in DRC, where we expect more oedematous malnutrition. Furthermore, this is an individually randomized trial that will monitor outcomes for an extended period, 4-5 months, after treatment. It also includes a group of children rarely studied but

For peer review only - http://bmjopen.bmj.com/site/about/guidelines.xhtml

common in everyday practice: MAM children who do not receive supplemental food. This study design should allow for robust conclusions not only regarding the non-inferiority of OptiMA compared to a typical standard SAM protocol but also an evaluation of sustained health and nutrition status over a longer period than previous trials.

Although the OptiMA simplified strategy is mostly MUAC-based, we chose to also include children with SAM according to the WHO definition based on oedema, MUAC, or WHZ. The stratified randomisation of children with SAM according to the WHO definition will shed some light on the clinical effects of the OptiMA strategy for this specific category of children. Moreover, this inclusion strategy will help to determine the segment of children with SAM defined by WHZ-only who were not identified through MUAC with a threshold at 125 mm, in the context of the Kasaï province where stunting prevalence in children is higher than anywhere else in the DRC (35).

Trial status

The recruitment phase began in July 2019 and ended 22th January 2020. The estimated end date for this study is September 2020.

Abbreviations

ALIMA: Alliance for International Medical Action ; AM: acute malnutrition; CNES: Comité National d'Ethique de la Santé); DRC: Democratic Republic of Congo; DSMB: data safety monitoring board; MAM: moderate acute malnutrition; MoH: Ministry of Health; MUAC: Mid Upper Arm Circumference; OptiMA: Optimizing treatment for Acute Malnutrition; RUSF: ready to use therapeutic food ; RUTF: ready-to-use therapeutic food; SAM: severe acute malnutrition; SD: standard deviation; UN: united nations ; WHO: World Health Organization; WHZ: weight-for-height Z-score.

Declarations

Ethics approval

The study is conducted in accordance with the Declaration of Helsinki. The protocol has been approved by the DRC national ethics committee (CNES) (94/CNES/BN/PMMF/2018) and by the Ethics Evaluation Committee of Inserm, the French National Institute for Health and Medical Research (Comité d'Evaluation Ethique de l'Inserm) (18-545). The final version of the protocol, version 3.1, dated 22 October 2019, is available for sharing on request, and included three minor amendments to protocol version 2.0 (dated 6 March 2019) and version 3.0 (dated 18 April 2019), which was shared with national and international ethical committees and did not require a new authorization. In November 2019, the CNES performed a field visit at

For peer review only - http://bmjopen.bmj.com/site/about/guidelines.xhtml

each of the study sites and then renewed approval (152/CNES/BN/PMMF/2019) for the continuation of the trial.

Consent for publication

Not required.

Data statement and sharing

Plan to share data and material is available on clinical.trial.gov registration. Individual participant data that underlie the results reported in published article, after deidentification (text, tables, figures, and appendices) will be accessible on use controlled access repositories. Authorship eligibility for dissemination results will be in line with the International Committee of Medical Journal Editors' recommendations.

Competing interests

KP serves on the Social Purposes Advisory Commission of Nutriset, a main producer of lipid-based nutrient supplement products. No other interests are declared.

Funding statement

The study is supported by the Innocent Foundation (London, UK) and received additional funding from European Commission through the European Civil Protection and Humanitarian Aid Operations (ECHO, Brussels, Belgium), grant number ECHO/COD/BUD/2018/91023. The funders had no role in study design, data collection and analysis, decision to publish, or preparation of the manuscript.

This document covers humanitarian aid activities implemented with the financial assistance of the European Union. The views expressed herein should not be taken, in any way, to reflect the official opinion of the European Union, and the European Commission is not responsible for any use that can be made of the information it contains.

Authors contributions

SS and RB developed the clinical (SS) and methodological (RB) study concept. RB, CC, XA, DG, AA, KP and SS designed the study methodology and wrote the protocol. CC, VH, HB and MK coordinate the study teams. LIB, GTS coordinate the Ministry of Health staff working in the trial. CC, VH, HB, LIB, GTS, AB, CY and RB organize and supervise data collection. CC, VH, DG, XA, SS and RB developed the statistical analysis strategy. CC wrote the first draft of the manuscript. SS and RB were primarily responsible for the final content of the manuscript. CY created the software tool for randomization and developed the database. All authors critically reviewed the first draft and had a substantial writing contribution to the development of the final manuscript.

For peer review only - http://bmjopen.bmj.com/site/about/guidelines.xhtml
For peer review only

Acknowledgements

We are indebted to the women and children who participate in the study. The authors wish to particularly acknowledge the ALIMA operational team on the ground who ran the study on a day-to-day basis. The authors also acknowledge the Ministry of Health of the Democratic Republic of Congo through the commitment of key representatives from the Kamuesha health zone and the National Nutrition Programme. We acknowledge the implementing partners of the inter-agency Nutrition Cluster Kasaï Province, in particular representatives from UNICEF and the World Food Programme. We also acknowledge Maguy Daures (Inserm UMR 1219, Bordeaux), Valérie Journot (Inserm UMR 1219, Bordeaux) and Claire Levy-Marchal (ALIMA, Paris) for having advising the study team on the trial methodology.

This study was developed as part of the Clinical and Operational Research Alliance (CORAL). This research platform aims at developing high-quality innovative and transformative research programs within a partnership between scientists from the research institute Inserm (Institut National de la Santé et la Recherche Médicale, Bordeaux and Abidjan) and the humanitarian organization ALIMA (Alliance for International Medical Action), primarily on improving maternal and child health outcomes. A board of directors defines the scientific policy of the CORAL partnership alongside with the supervision of research projects and dissemination of results. It consists of senior representatives from both ALIMA and Inserm: Renaud Becquet (Inserm Bordeaux, methodological co-chair), Susan Shepherd (ALIMA, medical co-chair), Augustin Augier (ALIMA), Moumouni Kinda (ALIMA), Marie Jaspard (ALIMA & Inserm Abidjan), Claire Levy-Marchal (ALIMA), and Xavier Anglaret (Inserm Bordeaux & Abidjan). The CORAL research platform meets annually with an external scientific advisory board to review projects and strategic orientation.

Finally, we warmly acknowledge the members of the Data Safety Monitoring Board (DSMB) for the OptiMA-DRC trial: Yves Martin-Prevel (DSMB chair, French National Institute for Sustainable Development (IRD), NUTRIPASS team, University of Montpellier, Montpellier, France), Matthew Colderon (Epicentre, New York, USA) and Katia Castetbon (DSMB statistician, School of Public Health, Université Libre de Bruxelles, Bruxelles, Belgium).

References

1. Levels and trends in child malnutritionUNICEF / WHO / World Bank Group Joint Child Malnutrition EstimatesKey findings of the 2019 edition [Internet]. [cité 18 nov 2019]. Disponible sur: https://www.who.int/nutgrowthdb/jme-2019-key-findings.pdf?ua=1

For peer review only - http://bmjopen.bmj.com/site/about/guidelines.xhtml

2. Black RE, Victora CG, Walker SP, Bhutta ZA, Christian P, de Onis M, et al. Maternal and child undernutrition and overweight in low-income and middle-income countries. The Lancet. 3 août 2013;382(9890):427‑51.

3. Isanaka S, Boundy EO, Grais RF, Myatt M, Briend A. Improving Estimates of Numbers of Children With Severe Acute Malnutrition Using Cohort and Survey Data. Am J Epidemiol. 15 déc 2016;184(12):861‑9.

4. Hure A, Oldmeadow C, Attia J. Invited Commentary: Improving Estimates of Severe Acute Malnutrition Requires More Data. Am J Epidemiol. 15 déc 2016;184(12):870‑2.

5. Unicef. UNICEF DRC Humanitarian Situation Report March, 2018 - Democratic Republic of the Congo [Internet]. ReliefWeb. [cité 16 janv 2020]. Disponible sur: https://reliefweb.int/report/democratic-republic-congo/unicef-drc-humanitarian-situation-report-march-2018

6. WHO child growth standards and the identification of severe acute malnutrition in infants and children - A Joint Statement by the World Health Organization and the United Nations Children's Fund- 2009 [Internet]. [cité 16 janv 2020]. Disponible sur: https://apps.who.int/iris/bitstream/handle/10665/44129/9789241598163_eng.pdf;jsessionid=7C1A602ED39C4B7995CBE93E690CA58C?sequence=1

7. World Health Organization, Nutrition for Health and Development. Guideline. [Internet]. 2013 [cité 16 janv 2020]. Disponible sur: http://www.ncbi.nlm.nih.gov/books/NBK190328/

8. Collins S, Sadler K, Dent N, Khara T, Guerrero S, Myatt M, et al. Key Issues in the Success of Community-Based Management of Severe Malnutrition. Food Nutr Bull. sept 2006;27(3_suppl3):S49‑82.

9. No Wasted Lives Brochure 2018 [Internet]. [cité 18 nov 2019]. Disponible sur: https://static1.squarespace.com/static/58da81cdd1758e39ca705526/t/5a7adf8171c10b8aa3b4462f/1518002053092/No+Wasted+Lives+Brochure+2018.pdf

10. Coalition No Wasted Lives. Etat de la malnutrition [Internet]. État de la malnutrition aiguë. [cité 13 janv 2020]. Disponible sur: https://acutemalnutrition.org/fr/countries

11. Horton, Susan, Shekar, Meera, McDonald, Christine, Mahal, Ajay, Krystene Brooks, Jana. Scaling Up Nutrition : What Will it Cost? [Internet]. The International Bank for Reconstruction and Development / The World Bank; 2010. Disponible sur: http://hdl.handle.net/10986/2685

12. Rogers E, Myatt M, Woodhead S, Guerrero S, Alvarez JL. Coverage of community-based management of severe acute malnutrition programmes in twenty-one countries, 2012-2013. PloS One. 2015;10(6):e0128666.

13. Isanaka S, Menzies NA, Sayyad J, Ayoola M, Grais RF, Doyon S. Cost analysis of the treatment of severe acute malnutrition in West Africa. Matern Child Nutr [Internet]. oct 2017 [cité 18 nov 2019];13(4). Disponible sur: https://onlinelibrary.wiley.com/doi/abs/10.1111/mcn.12398

14. Briend A, Maire B, Fontaine O, Garenne M. Mid-upper arm circumference and weight-for-height to identify high-risk malnourished under-five children. Matern Child Nutr. janv 2012;8(1):130‑3.

For peer review only

For peer review only - http://bmjopen.bmj.com/site/about/guidelines.xhtml

15. Binns P, Dale N, Hoq M, Banda C, Myatt M. Relationship between mid upper arm circumference and weight changes in children aged 6–59 months. Arch Public Health. déc 2015;73(1):1‑10.

16. Roberfroid D, Hammami N, Lachat C, Weise Prinzo Z, Sibson V, Guesdon B, et al. Utilization of mid-upper arm circumference versus weight-for-height in nutritional rehabilitation programmes: a systematic review of evidence. 2013 [cité 13 janv 2020]; Disponible sur: http://hdl.handle.net/1854/LU-5700355

17. Burza S, Mahajan R, Marino E, Sunyoto T, Shandilya C, Tabrez M, et al. Community-based management of severe acute malnutrition in India: new evidence from Bihar. Am J Clin Nutr. avr 2015;101(4):847‑59.

18. Alé FGB, Phelan KPQ, Issa H, Defourny I, Le Duc G, Harczi G, et al. Mothers screening for malnutrition by mid-upper arm circumference is non-inferior to community health workers: results from a large-scale pragmatic trial in rural Niger. Arch Public Health [Internet]. déc 2016 [cité 18 nov 2019];74(1). Disponible sur: http://archpublichealth.biomedcentral.com/articles/10.1186/s13690-016-0149-5

19. Goossens S, Bekele Y, Yun O, Harczi G, Ouannes M, Shepherd S. Mid-upper arm circumference based nutrition programming: evidence for a new approach in regions with high burden of acute malnutrition. PloS One. 2012;7(11):e49320.

20. Shekar M, Kakietek J, Dayton Eberwein J, Walters D. An Investment Framework for Nutrition: Reaching the Global Targets for Stunting, Anemia, Breastfeeding, and Wasting [Internet]. The World Bank; 2017 [cité 21 nov 2019]. Disponible sur: http://elibrary.worldbank.org/doi/book/10.1596/978-1-4648-1010-7

21. Maust A, Koroma AS, Abla C, Molokwu N, Ryan KN, Singh L, et al. Severe and Moderate Acute Malnutrition Can Be Successfully Managed with an Integrated Protocol in Sierra Leone. J Nutr. 1 nov 2015;145(11):2604‑9.

22. Kangas ST, Salpéteur C, Nikièma V, Talley L, Ritz C, Friis H, et al. Impact of reduced dose of ready-to-use therapeutic foods in children with uncomplicated severe acute malnutrition: A randomised non-inferiority trial in Burkina Faso. Persson LÅ, éditeur. PLOS Med. 27 août 2019;16(8):e1002887.

23. Daures M, Phelan K, Issoufou M, Kouanda S, Sawadogo O, Issaley K, et al. New approach to simplifying and optimizing acute malnutrition treatment in children aged 6 to 59 months: the OptiMA single-arm proof-of-concept trial in Burkina Faso. Br J Nutr. 10 déc 2019;1‑31.

24. Community Based Management of Sever Acute Malnutrition - A Joint Statement by the World Health Organization, the World Food Programme, the United Nations System Standing Committee on Nutrition and the United Nations Children's Fund - 2007 [Internet]. [cité 18 nov 2019]. Disponible sur: https://www.unicef.org/publications/files/Community_Based_Management_of_Sever_Acute__Malnutirtion.pdf

25. Chan A-W, Tetzlaff JM, Altman DG, Laupacis A, Gøtzsche PC, Krleža-Jerić K, et al. SPIRIT 2013 statement: defining standard protocol items for clinical trials. Ann Intern Med. 5 févr 2013;158(3):200‑7.

For peer review only

For peer review only - http://bmjopen.bmj.com/site/about/guidelines.xhtml

26. DR Congo - Situation Report N°14 : Complex Emergency in the Kasai Region (23 octobre 2017) | HumanitarianResponse [Internet]. [cité 20 mars 2020]. Disponible sur: https://www.humanitarianresponse.info/en/operations/democratic-republic-congo/document/dr-congo-situation-report-n%C2%B014-complex-emergency-kasai

27. O'Sullivan NP, Lelijveld N, Rutishauser-Perera A, Kerac M, James P. Follow-up between 6 and 24 months after discharge from treatment for severe acute malnutrition in children aged 6-59 months: A systematic review. PLOS ONE. 30 août 2018;13(8):e0202053.

28. Grellety E, Babakazo P, Bangana A, Mwamba G, Lezama I, Zagre NM, et al. Effects of unconditional cash transfers on the outcome of treatment for severe acute malnutrition (SAM): a cluster-randomised trial in the Democratic Republic of the Congo. BMC Med. 26 2017;15(1):87.

29. James P, Sadler K, Wondafrash M, Argaw A, Luo H, Geleta B, et al. Children with Moderate Acute Malnutrition with No Access to Supplementary Feeding Programmes Experience High Rates of Deterioration and No Improvement: Results from a Prospective Cohort Study in Rural Ethiopia. PloS One. 2016;11(4):e0153530.

30. Bailey J, Lelijveld N, Marron B, Onyoo P, Ho LS, Manary M, et al. Combined Protocol for Acute Malnutrition Study (ComPAS) in rural South Sudan and urban Kenya: study protocol for a randomized controlled trial. Trials [Internet]. déc 2018 [cité 18 nov 2019];19(1). Disponible sur: https://trialsjournal.biomedcentral.com/articles/10.1186/s13063-018-2643-2

31. Bahwere P, Balaluka B, Wells JCK, Mbiribindi CN, Sadler K, Akomo P, et al. Cereals and pulse-based ready-to-use therapeutic food as an alternative to the standard milk- and peanut paste-based formulation for treating severe acute malnutrition: a noninferiority, individually randomized controlled efficacy clinical trial. Am J Clin Nutr. avr 2016;103(4):1145‑61.

32. Bahwere P, Banda T, Sadler K, Nyirenda G, Owino V, Shaba B, et al. Effectiveness of milk whey protein-based ready-to-use therapeutic food in treatment of severe acute malnutrition in Malawian under-5 children: a randomised, double-blind, controlled non-inferiority clinical trial. Matern Child Nutr. juill 2014;10(3):436‑51.

33. Moher D, Hopewell S, Schulz KF, Montori V, Gøtzsche PC, Devereaux PJ, et al. CONSORT 2010 Explanation and Elaboration: updated guidelines for reporting parallel group randomised trials. BMJ [Internet]. 24 mars 2010 [cité 17 janv 2020];340. Disponible sur: https://www.bmj.com/content/340/bmj.c869

34. Lelijveld N, Bailey J, Mayberry A, Trenouth L, N'Diaye DS, Haghparast-Bidgoli H, et al. The « ComPAS Trial » combined treatment model for acute malnutrition: study protocol for the economic evaluation. Trials. 24 avr 2018;19(1):252.

35. Kismul H, Acharya P, Mapatano MA, Hatløy A. Determinants of childhood stunting in the Democratic Republic of Congo: further analysis of Demographic and Health Survey 2013–14. BMC Public Health. 1 août 2017;18(1):74.

For peer review only

For peer review only - http://bmjopen.bmj.com/site/about/guidelines.xhtml

Figure 1: Schedule of enrolment, interventions and assessments OptiMA-DRC overview

		STUDY PERIOD							
	Enrolment	Allocation	Post-allocation						Close-out
TIMEPOINT		d0	m_1	m_2	m_3	m_4	m_5	m_6	m_6
**ENROLMENT:**									
**Eligibility screening***	X								
		Outpatient visit							
**Informed consent**		X							
**Allocation**		X							
**INTERVENTIONS:**		X	Outpatient or home visits **						
**Standard strategy**		X	X	X	X	X	X	X	
**OptiMA strategy**		X	X	X	X	X	X	X	
**ASSESSMENTS:**									
**Clinical examination**		X	X	X	X	X	X	X	X
**Anthropometry measurements**	X	X	X	X	X	X	X	X	X
			Inpatient wards						
**Hospitalization if required**			X	X	X	X	X	X	X

For peer review only

d=day; m=month

*Monthly active screening in 60 villages and passive screening during outpatient visit in 4 health centre

**Weekly (for those living in villages at 14 km or less from the health centre) or bimonthly (for those living in villages more than 14 km from the health centre) outpatient visits at health centre for participants with RUTF supplementation

*** Bimonthly home visits for children without RUTF supplementation.

Additional file 1. Standard operating procedure for taking anthropometric measurements

OptiMA study – The Democratic Republic of Congo

ALIMA	République Démocratique du Congo Ministère de la Santé; Inserm	SOP_01_OptiMA-RDC	
	Procedure for taking anthropometric measurements	Version	
		V1.0	24/05/2019

Version		Modification
1.0	24/05/2019	Creation

For peer review only

Objective

This document is intended to describe the process of taking anthropometric measurements in the OptiMA -DRC clinical trial.

Scope of application

These procedures are intended to be used by the research team of the OptiMA-DRC trial, and more specifically for the nurses and community health workers to follow during the study.

Acronyms

AM: Acute Malnutrition

CHW: Community Health Workers

CMAM: Community Management of Acute Malnutrition

CTT: Clinical Trial Technician

IT: Titular Nurse of health centre

MUAC: Mid Upper Arm Circumference

WHZ: Weight for Height Z score

1. General principles

From the start of the inclusion phase of the trial, research teams will organize monthly active screening for acute malnutrition (AM) in villages in the study health zones. During these sessions, the community health workers (CHWs) previously trained for this purpose will take anthropometric measurements (weight, height, mid upper arm circumference (MUAC)) and check for bilateral nutritional oedema under the supervision of a nurse among all children aged 6 to 59 months present on the screening day and accompanied by a legal representative.

Children coming for a paediatric consultation at the health centre will also be screened for acute malnutrition by health centre staff using this procedure.

Anthropometric measurements will also be taken in the children included in the study during weekly follow-up visits to the health centre and during biweekly follow-up home visits.

2. Logistics/data collection

The equipment needed to take anthropometric measurements are:

- 25 kg Salter-type scale graduated to the nearest hundredth gram;
- A standard 5 kg weight;
- Height board;
- MUAC bracelets;
- Basins for weighing and soaps for cleaning the basins;
- Registry dedicated to active screening for AM in villages as part of the OptiMA-DRC trial;
- Dedicated registry for passive AM screening in health centres as part of the OptiMA-DRC trial;
- Therapeutic follow-up sheet used for children in the study at the inclusion visit.

3. Organization of activities

Concerning active screening in the villages and home follow-up visits, the nurse will travel by motorbike to the villages in his or her health zone according to the planned tours. He will bring a Salter scale, a 5 kg weight, a height board, the register for active screening or the therapeutic sheets for home follow-up visits.

The basins will be placed in each health area by axis, according to the geographical position and distance between villages. After each daily activity, the CHWs will proceed to clean the basin.

At the level of the health centre, the health and research staff will ensure that the equipment is available and functional on a daily basis.

4. Techniques

<u>Weight:</u>

Tare the scale before weighing the 5 kg weight. Repeat the tare on the scale every 10 children weighed.

To check the accuracy of the scale:

- Put the standard 5kg weight on the scale;
- If the scale does not read '5kg', adjust the scale when empty until it gives the correct weight of 5kg.

The plastic basin attached with four cords should be close to the floor for the child to feel safe. The use of the basin is comfortable for children and more hygienic because it can be easily washed.

The weight of the child (to the nearest 100 grams) is measured using the 25 kg Salter scale attached to the basin, following these steps:

- The scale must not be exposed to the sun;

For peer review only

- Readjust the scale to zero before each weighing;
- Explain to the mother that her child will be weighed naked in the basin to get her real weight and ask her to take off the child's clothes;
- The mother, helped by the CHW, places the child in the basin and remains next to the basin so that the child doesn't get too agitated and to reassure him or her;
- When the child does not move anymore, read the weight to the nearest 100 g (the needle should be at eye level) and note the weight in the register.

Height:

The height will be measured to the nearest 0.1 cm with a 130 cm wooden height board with graduated mm markings on each side. It will be measured in the prone position for children under 87 cm and in the standing position for children 87 cm or taller.

Children measuring less than 87 cm (prone position, see illustration below):

- Explain the procedure to the mother and ask her to remove the child's shoes and any ornaments in the child's hair.
- Place the board flat on the floor. The child lies down in the middle of the scale with the mother's help, with the child's feet on the side of the slider.
- The assistant holds the child's head between her/his hands at ear level and holds it firmly against the fixed part of the scale, the child's hair should be compressed. The child looks straight ahead.
- The measurer places his or her hands above the child's ankles, gently stretches the legs and places one hand on the child's thighs to prevent the child from bending his or her legs.
- Holding the legs firmly, he or she pushes the slider firmly flat against the soles of the child's feet.
- To read the measurement, the slider must be perpendicular to the axis of the scale and vertical. He then reads to the nearest 0.1cm and notes in the register.

Children measuring 87 cm or more (standing position) (see illustration below):

- Explain the procedure to the mother and ask her to remove the child's shoes and any ornaments in the hair;
- Place the standing scale on a solid flat surface and if possible against a wall.
- The child stands upright in the middle of the height board and touches the vertical plane of the board.
- The assistant holds the head, shoulders, buttocks, knees, ankles against the scale while the measurer positions the head and slide. He then reads to the nearest 0.1cm and notes in the register.

Calculating the weight for height Z-score

The weight for height Z-score (WHZ) should be recorded according to the weight for height Z-score table, which is from the national CMAM protocol (2016) and was used in the OptiMA trial. The nurse refers to the weight (in kg) and height (in cm) measured to read the child's WHZ.

How to use the weight/height ratio table?

Example: a child is 63 cm tall and weighs 6.5 kg.

For peer review only

- Take the table, look at the first column and search for the number 63cm (=height).
- Take a ruler or sheet of paper and place it under the number 63. On this line, find the weight corresponding to the child (in this case 6.5).
- Move up the corresponding column to determine which column it is. For this example, it corresponds to the column MEDIAN WEIGHT. For this example, the child's weight is normal in relation to his or her height. Therefore, the child's weight is appropriate for his or her height.

Example: a child is 78 cm tall and weighs 8.3 kg. This child is between column -2 and -3 Z-score or between MAM and SAM. He is too thin for his height; he is <-2 (less) and >-3 (more): he has MODERATE MALNUTRITION but NOT severe malnutrition. Record the WHZ as "<-2" in the register.

NOTE: It may happen that the height is not a round number, in which case rounding should be done according to the pattern below. For example: the size 80.1 and 80.2 are rounded to 80.0; 80.3, 80.4, 80.6 and 80.7 are rounded to 80.5; 80.8 and 80.9 are rounded to 81.0.

<u>The Mid Upper Arm Circumference (MUAC):</u>

- Ask the mother to remove the clothing that covers the child's left arm. Make a mark halfway between the shoulder and the left elbow (middle of the left arm). To do this, take a piece of string (or the MUAC itself), and place one end of the string on top of (arrow 1) and the other end on the elbow (arrow 2), making sure the string is taut. Then bend the string in half, bringing the end of the elbow back towards the end of the shoulder to the point halfway between the shoulder and the elbow.
- Another method can be used. Place the 0 of the MUAC (indicated by the 2 arrows) on top of the shoulder (arrow 4) and bring the other end towards the elbow (arrow 5). Read the number on the elbow to the nearest centimetre.
- Divide this number by two to get an estimate of the midpoint between the shoulder and the elbow. Make a mark on the arm with a pen at this value (arrow 6).
- Release the child's arm and place the MUAC around the arm at the mark. Make sure that the numbers are right side up. Make sure that the MUAC touches the skin (arrow 7).
- Check the tension on the MUAC. Make sure that the tension is correct, i.e. that the MUAC is not too tight (arm compressed) or too loose (the MUAC bracelet is not touching the skin all around the arm) (arrows 7 and 8).
- Repeat each step if necessary.
- When the MUAC is correctly positioned and the tension applied is good, read and pronounce the measurement out loud to the nearest 0.1cm (arrow 10).
- Record the measurement immediately.

<u>Checking for Nutritional Oedema:</u>

Nutritional oedema is detected in the following way:

- Normal pressure is exerted with the thumb on both feet for at least three seconds.
- If the thumb impression persists on both feet after removing the thumbs, then the child has oedema.
- Only children with bilateral oedema are recorded as having oedema.

For peer review only

You have to test with the pressure of your thumb! It is not enough just to look!

**Severity of Oedema**	**Coding**
**Mild Oedema**: present in both feet	**+**
**Moderate Oedema**: present in both feet and the lower part of both legs, and both hands and the lower part of both forearms. Intermediate between mild and severe oedema ++	**++**
**Severe Oedema:** generalized, including both feet, legs, hands, arms and face	**+++**

For peer review only

Additional file 2: RUTF ration per week according to Standard and OptiMA strategy

Standard RUTF ration per week		OptiMA RUTF ration per week			
	MUAC <115 or oedema or WHZ < -3 Z score		MUAC < 115 mm or oedema	MUAC [115-119 mm]	MUAC >=120 mm
Weight (KG)	Number of RUTF sachet per week	Weight (KG)	Number of RUTF sachet per week		
**3.0-3.4**	9	**3.0-3.4**	10	8	7
**3.5 - 4.9**	11	**3.5 - 4.0**	11	8	7
		**4.1 - 4.4**	12	9	7
		**4.5 - 4.9**	13	10	7
**5.0 - 6.9**	14	**5.0 - 5.4**	14	11	7
		**5.5 - 5.9**	15	12	7
		**6.0 - 6.4**	16	12	7
		**6.5 - 6.9**	17	13	7
**7.0 - 9.9**	21	**7.0 -7.4**	18	13	8
		**7.5 - 7.9**	19	14	8
		**8.0 - 8.4**	20	15	9
		**8.5 - 8.9**	22	15	9
		**9.0 - 9.4**	23	16	9
		**9.5 - 9.9**	24	17	9
**10.0 - 14.9**	28	**10.0 - 10.4**	25	18	10
		**10.5 - 10.9**	26	19	10
		**11.0 -11.4**	27	20	10
		**11.5 - 11.9**	29	21	10
		**12.0 - 12.4**	30	22	11
		**12.5 - 12.9**	31	22	11
		**13.0 - 13.4**	33	23	12
		**13.5 - 13.9**	34	24	12
		**14.0 - 14.4**	35	25	12
		**14.5 -14.9**	36	26	13
**15.0 - 19.9**	35	**15.0 - 15.4**	36	28	14
		**15.5 - 15.9**	36	28	14
		**16.0 - 16.4**	36	28	14
		**16.5 - 16.9**	36	28	14
		**17.0 - 17.4**	36	28	14
		**17.5 - 17.9**	36	28	14
		**18.0 - 18.4**	36	28	14
		**18.5 - 18.9**	36	28	14
		**19.0 - 19.4**	36	28	14
		**19.5 - 19.9**	36	28	14

For peer review only

SPIRIT Checklist for OptiMA-DRC protocol submission for publication

		Reporting Item	Page Number
**Administrative information**			
Title	#1	Descriptive title identifying the study design, population, interventions, and, if applicable, trial acronym	1
Trial registration	#2a	Trial identifier and registry name. If not yet registered, name of intended registry	2
Trial registration: data set	#2b	All items from the World Health Organization Trial Registration Data Set	2
Protocol version	#3	Date and version identifier	13
Funding	#4	Sources and types of financial, material, and other support	13
Roles and responsibilities: contributorship	#5a	Names, affiliations, and roles of protocol contributors	13
Roles and responsibilities: sponsor contact information	#5b	Name and contact information for the trial sponsor	1
Roles and responsibilities: sponsor and funder	#5c	Role of study sponsor and funders, if any, in study design; collection, management, analysis, and interpretation of data; writing of the report; and the decision to submit the report for publication, including whether they will have ultimate authority over any of these activities	11
Roles and responsibilities: committees	#5d	Composition, roles, and responsibilities of the coordinating centre, steering committee, endpoint adjudication committee, data management team, and other individuals or groups overseeing the trial, if applicable (see Item 21a for data monitoring committee)	11, 14
**Introduction**			
Background and rationale	#6a	Description of research question and justification for undertaking the trial, including summary of relevant studies (published and unpublished) examining benefits and harms for each intervention	3,4,5
Background and rationale: choice of comparators	#6b	Explanation for choice of comparators	4
Objectives	#7	Specific objectives or hypotheses	5

For peer review only

Trial design	#8	Description of trial design including type of trial (eg, parallel group, crossover, factorial, single group), allocation ratio, and framework (eg, superiority, equivalence, non-inferiority, exploratory)	5
**Methods: Participants, interventions, and outcomes**			
Study setting	#9	Description of study settings (eg, community clinic, academic hospital) and list of countries where data will be collected. Reference to where list of study sites can be obtained	6
Eligibility criteria	#10	Inclusion and exclusion criteria for participants. If applicable, eligibility criteria for study centres and individuals who will perform the interventions (eg, surgeons, psychotherapists)	6,7
Interventions: description	#11a	Interventions for each group with sufficient detail to allow replication, including how and when they will be administered	9,10
Interventions: modifications	#11b	Criteria for discontinuing or modifying allocated interventions for a given trial participant (eg, drug dose change in response to harms, participant request, or improving / worsening disease)	8,11
Interventions: adherance	#11c	Strategies to improve adherence to intervention protocols, and any procedures for monitoring adherence (eg, drug tablet return; laboratory tests)	10
Interventions: concomitant care	#11d	Relevant concomitant care and interventions that are permitted or prohibited during the trial	9
Outcomes	#12	Primary, secondary, and other outcomes, including the specific measurement variable (eg, systolic blood pressure), analysis metric (eg, change from baseline, final value, time to event), method of aggregation (eg, median, proportion), and time point for each outcome. Explanation of the clinical relevance of chosen efficacy and harm outcomes is strongly recommended	7,19,20
Participant timeline	#13	Time schedule of enrolment, interventions (including any run-ins and washouts), assessments, and visits for participants. A schematic diagram is highly recommended (see Figure)	12,21 (figure 1)
Sample size	#14	Estimated number of participants needed to achieve study objectives and how it was determined, including clinical and statistical assumptions supporting any sample size calculations	7
Recruitment	#15	Strategies for achieving adequate participant enrolment to reach target sample size	7,8
**Methods: Assignment of interventions (for controlled trials)**			

For peer review only

Allocation: sequence generation	#16a	Method of generating the allocation sequence (eg, computer-generated random numbers), and list of any factors for stratification. To reduce predictability of a random sequence, details of any planned restriction (eg, blocking) should be provided in a separate document that is unavailable to those who enrol participants or assign interventions	8
Allocation concealment mechanism	#16b	Mechanism of implementing the allocation sequence (eg, central telephone; sequentially numbered, opaque, sealed envelopes), describing any steps to conceal the sequence until interventions are assigned	8
Allocation: implementation	#16c	Who will generate the allocation sequence, who will enrol participants, and who will assign participants to interventions	8
Blinding (masking)	#17a	Who will be blinded after assignment to interventions (eg, trial participants, care providers, outcome assessors, data analysts), and how	10
Blinding (masking): emergency unblinding	#17b	If blinded, circumstances under which unblinding is permissible, and procedure for revealing a participant's allocated intervention during the trial	"n/a" no blinding
**Methods: Data collection, management, and analysis**			
Data collection plan	#18a	Plans for assessment and collection of outcome, baseline, and other trial data, including any related processes to promote data quality (eg, duplicate measurements, training of assessors) and a description of study instruments (eg, questionnaires, laboratory tests) along with their reliability and validity, if known. Reference to where data collection forms can be found, if not in the protocol	10
Data collection plan: retention	#18b	Plans to promote participant retention and complete follow-up, including list of any outcome data to be collected for participants who discontinue or deviate from intervention protocols	10
Data management	#19	Plans for data entry, coding, security, and storage, including any related processes to promote data quality (eg, double data entry; range checks for data values). Reference to where details of data management procedures can be found, if not in the protocol	8,10
Statistics: outcomes	#20a	Statistical methods for analysing primary and secondary outcomes. Reference to where other details of the statistical analysis plan can be found, if not in the protocol	11
Statistics: additional analyses	#20b	Methods for any additional analyses (eg, subgroup and adjusted analyses)	11

For peer review only

Statistics: analysis population and missing data	#20c	Definition of analysis population relating to protocol non-adherence (eg, as randomised analysis), and any statistical methods to handle missing data (eg, multiple imputation)	11
**Methods: Monitoring**			
Data monitoring: formal committee	#21a	Composition of data monitoring committee (DMC); summary of its role and reporting structure; statement of whether it is independent from the sponsor and competing interests; and reference to where further details about its charter can be found, if not in the protocol. Alternatively, an explanation of why a DMC is not needed	10
Data monitoring: interim analysis	#21b	Description of any interim analyses and stopping guidelines, including who will have access to these interim results and make the final decision to terminate the trial	11
Harms	#22	Plans for collecting, assessing, reporting, and managing solicited and spontaneously reported adverse events and other unintended effects of trial interventions or trial conduct	10,11
Auditing	#23	Frequency and procedures for auditing trial conduct, if any, and whether the process will be independent from investigators and the sponsor	11,13 (national ethic committee audit)
**Ethics and dissemination**			
Research ethics approval	#24	Plans for seeking research ethics committee / institutional review board (REC / IRB) approval	11,13
Protocol amendments	#25	Plans for communicating important protocol modifications (eg, changes to eligibility criteria, outcomes, analyses) to relevant parties (eg, investigators, REC / IRBs, trial participants, trial registries, journals, regulators)	13
Consent or assent	#26a	Who will obtain informed consent or assent from potential trial participants or authorised surrogates, and how (see Item 32)	11,12
Consent or assent: ancillary studies	#26b	Additional consent provisions for collection and use of participant data and biological specimens in ancillary studies, if applicable	"n/a", no ancillary study planned
Confidentiality	#27	How personal information about potential and enrolled participants will be collected, shared, and maintained in order to protect confidentiality before, during, and after the trial	11
Declaration of interests	#28	Financial and other competing interests for principal investigators for the overall trial and each study site	13
Data access	#29	Statement of who will have access to the final trial dataset, and disclosure of contractual agreements that limit such access for investigators	13

For peer review only

Ancillary and post trial care	#30	Provisions, if any, for ancillary and post-trial care, and for compensation to those who suffer harm from trial participation	"n/a" participants are covered by insurance of the trial
Dissemination policy: trial results	#31a	Plans for investigators and sponsor to communicate trial results to participants, healthcare professionals, the public, and other relevant groups (eg, via publication, reporting in results databases, or other data sharing arrangements), including any publication restrictions	11
Dissemination policy: authorship	#31b	Authorship eligibility guidelines and any intended use of professional writers	13, no use of professional writers planned
Dissemination policy: reproducible research	#31c	Plans, if any, for granting public access to the full protocol, participant-level dataset, and statistical code	13
**Appendices**			
Informed consent materials	#32	Model consent form and other related documentation given to participants and authorised surrogates	13
Biological specimens	#33	Plans for collection, laboratory evaluation, and storage of biological specimens for genetic or molecular analysis in the current trial and for future use in ancillary studies, if applicable	"n/a" no biological specimens collected

For peer review only

BMJ Open

Simplifying and optimizing management of acute malnutrition in children aged 6 to 59 months: study protocol for a community-based individually randomized-controlled trial in Kasaï, Democratic Republic of Congo

Journal:	*BMJ Open*
Manuscript ID	bmjopen-2020-041213.R1
Article Type:	Protocol
Date Submitted by the Author:	05-Oct-2020
Complete List of Authors:	CAZES, Cécile; University of Bordeaux College of Health Sciences, Inserm, French National Research Institute for Sustainable Development (IRD), Bordeaux Population Health Research Center, Team IDLIC, UMR 1219<br>PHELAN, Kevin; ALIMA, Alliance for International Medical Action<br>Hubert, Victoire; ALIMA, Alliance for International Medical Action<br>ALITANOU, Rodrigue; ALIMA, Alliance for International Medical Action<br>BOUBACAR, Harouna; ALIMA, Alliance for International Medical Action<br>IZIE BOZAMA, Liévin; Ministry of Health, National Nutrition Programme (PRONANUT)<br>TSHIBANGU SAKUBU, Gilbert; Kamuesha health zone in the Kasaï province, Ministry of Health<br>BEUSCART, Aurélie; University of Bordeaux College of Health Sciences, Inserm, French National Research Institute for Sustainable Development (IRD), Bordeaux Population Health Research Center, Team IDLIC, UMR 1219<br>YAO, Cyrille; University Hospital Center of Treichville, PACCI research programme<br>Gabillard, Delphine; University of Bordeaux College of Health Sciences, Inserm, French National Research Institute for Sustainable Development (IRD), Bordeaux Population Health Research Center, Team IDLIC, UMR 1219<br>KINDA, Moumouni; ALIMA, Alliance for International Medical Action<br>AUGIER, Augustin; ALIMA, Alliance for International Medical Action<br>ANGLARET, Xavier; University of Bordeaux College of Health Sciences, Inserm, French National Research Institute for Sustainable Development (IRD), Bordeaux Population Health Research Center, Team IDLIC, UMR 1219<br>Shepherd, Susan; ALIMA, Alliance for International Medical Action<br>Becquet, Renaud; University of Bordeaux College of Health Sciences, Inserm, French National Research Institute for Sustainable Development (IRD), Bordeaux Population Health Research Center, Team IDLIC, UMR 1219
**Primary Subject Heading**:	Paediatrics
Secondary Subject Heading:	Nutrition and metabolism

Keywords:	Nutrition < TROPICAL MEDICINE, PUBLIC HEALTH, Clinical trials < THERAPEUTICS, Paediatric pathology < PAEDIATRICS, Community child health < PAEDIATRICS, Protocols & guidelines < HEALTH SERVICES ADMINISTRATION & MANAGEMENT

SCHOLARONE™
Manuscripts

I, the Submitting Author has the right to grant and does grant on behalf of all authors of the Work (as defined in the below author licence), an exclusive licence and/or a non-exclusive licence for contributions from authors who are: i) UK Crown employees; ii) where BMJ has agreed a CC-BY licence shall apply, and/or iii) in accordance with the terms applicable for US Federal Government officers or employees acting as part of their official duties; on a worldwide, perpetual, irrevocable, royalty-free basis to BMJ Publishing Group Ltd ("BMJ") its licensees and where the relevant Journal is co-owned by BMJ to the co-owners of the Journal, to publish the Work in this journal and any other BMJ products and to exploit all rights, as set out in our licence.

The Submitting Author accepts and understands that any supply made under these terms is made by BMJ to the Submitting Author unless you are acting as an employee on behalf of your employer or a postgraduate student of an affiliated institution which is paying any applicable article publishing charge ("APC") for Open Access articles. Where the Submitting Author wishes to make the Work available on an Open Access basis (and intends to pay the relevant APC), the terms of reuse of such Open Access shall be governed by a Creative Commons licence – details of these licences and which Creative Commons licence will apply to this Work are set out in our licence referred to above.

Other than as permitted in any relevant BMJ Author's Self Archiving Policies, I confirm this Work has not been accepted for publication elsewhere, is not being considered for publication elsewhere and does not duplicate material already published. I confirm all authors consent to publication of this Work and authorise the granting of this licence.

For peer review only

Simplifying and optimizing management of acute malnutrition in children aged 6 to 59 months: study protocol for a community-based individually randomized-controlled trial in Kasaï, Democratic Republic of Congo

Running title: OptiMA-DRC

Cécile CAZES [1], Kevin PHELAN [2], Victoire HUBERT [3], Rodrigue ALITANOU [3], Harouna BOUBACAR [3], Liévin IZIE BOZAMA [4], Gilbert TSHIBANGU SAKUBU [5], Aurélie BEUSCART [1], Cyrille YAO [6], Delphine GABILLARD [1], Moumouni KINDA [7], Augustin AUGIER [2], Xavier ANGLARET [1], Susan SHEPHERD [7], Renaud BECQUET [1]

1. University of Bordeaux, Inserm, French National Research Institute for Sustainable Development (IRD), Bordeaux Population Health Research Center, Team IDLIC, UMR 1219, Bordeaux, France
2. The Alliance for International Medical Action (ALIMA), Paris, France
3. The Alliance for International Medical Action (ALIMA), Kamuesha, Democratic Republic of Congo
4. National Nutrition Programme (PRONANUT), Ministry of Health, Kinshasa, Democratic Republic of Congo
5. Kamuesha health zone in the Kasaï province, Ministry of Health, Kamuesha, Democratic Republic of Congo
6. PACCI research programme, University Hospital of Treichville, Abidjan, Ivory Coast
7. The Alliance for International Medical Action (ALIMA), Dakar, Senegal

Address for correspondence:

Cécile CAZES, MPH

Bordeaux Population Health Research Center – Team IDLIC

University of Bordeaux

146 rue Léo Saignat,

33000, Bordeaux, France

Email: cecile.cazes@u-bordeaux.fr

Contact and address of the trial sponsor:

Plateforme CORAL - Clinical & Operational Research Alliance

ALIMA - The Alliance for International Medical Action, Dakar, Sénégal et

Inserm- U1219 Bordeaux Population Health, Université de Bordeaux, France

15 rue des immeubles industriels 75011 Paris

Email : claire.levy-marchal@coral.alima.ngo

For peer review only - http://bmjopen.bmj.com/site/about/guidelines.xhtml

Word count: 3841 words (from background to discussion)

Abstract

Introduction

Acute malnutrition (AM) is a continuum condition, arbitrarily divided into moderate and severe (SAM) categories, funded and managed in separate programs under different protocols. Optimizing acute MAlnutrition treatment (OptiMA) aims to simplify and optimize AM management by treating children with mid-upper arm circumference (MUAC) <125 mm or edema with one product – ready-to-use therapeutic food – at a gradually tapered dose. Our main objective was to compare the OptiMA strategy with the standard nutritional protocol in children 6-59 months presenting with MUAC <125 mm or oedema without additional complications, as well as in children classified as uncomplicated SAM (i.e. MUAC <115 mm or WHZ <-3 or with oedema).

Methods and Analysis

This study was a non-inferiority, individually randomized controlled clinical trial conducted at community level in the Democratic Republic of Congo. Children 6-59 months presenting with MUAC < 125 mm or WHZ <-3 or with bipedal oedema and without medical complication were included after signed informed consent in outpatient health facilities. All participants are followed for 6 months. Success in both arms is defined at 6-months post inclusion as being alive, not acutely malnourished per the definition applied at inclusion, and without an additional episode of acute malnutrition throughout the 6-month observation period. Recovery among children with uncomplicated SAM is the main secondary outcome. For the primary objective, 890 participants were needed, and 480 children with SAM were needed for the main secondary objective. We will perform non-inferiority analyses in per-protocol and intention-to-treat for both outcomes.

Ethics and dissemination

Ethics approvals were obtained from the National Health Ethics Committee of the Democratic Republic of Congo and from the Ethics Evaluation Committee of Inserm, the French National Institute for Health and Medical Research (Paris, France). We will submit results for publication to a peer-reviewed journal and disseminate findings in international and national conferences and meetings.

Trial registration: NCT03751475. Registered 19 September 2018, https://clinicaltrials.gov/ct2/show/NCT03751475

Keywords: Acute malnutrition, child, MUAC, RUTF, Africa, clinical trial, protocol

For peer review only - http://bmjopen.bmj.com/site/about/guidelines.xhtml

Strengths and limitations of this study

- This is the first study of a MUAC-based malnutrition treatment protocol with gradually tapered dose of RUTF that uses an individually randomized control design.
- The main outcome takes into account sustained health and nutrition status at 6 months after inclusion, including post-recovery relapse, and spontaneous recovery for children who are not eligible for RUTF supplementation.
- The methodology used for randomization (i.e. stratification based on WHO definition of SAM) allows for two robust analyses: first, how all children with MUAC <125 mm or WHZ <-3 or oedema respond under OptiMA; and second, how children only meeting the WHO definition of SAM respond.
- This trial is not a multisite study and only takes place in one country, the Democratic Republic of Congo.

Background

Acute malnutrition (AM) affects an estimated 50 million children under five years of age, including 16.6 million severe cases (SAM), and is an underlying cause of 800,000 deaths each year worldwide (1,2). Other estimation methods based on incidence instead of prevalence suggest the caseload for this major public health concern may be substantially higher (3,4). One quarter of all acutely malnourished children are in Africa and as many as 2 million severely wasted children were reported in the Democratic Republic of Congo (DRC) in 2018 (5).

Since the publication of the United Nations (UN) Joint Statement in 2009, millions of children have received treatment for SAM via standard community-based management (6,7) with >80% of children recovering in outpatient care (8). Despite more than a decade of expanded access to treatment, coverage of nutrition programs remains remarkably low, as few as 20% of wasted children actually receive treatment (9,10) and the World Bank estimates that it would take an additional US$6.3 billion per year to reach a coverage of 80% (11). There is therefore an urgent need to better allocate available resources through the simplification and optimization of current acute malnutrition protocols. Adapting programming in three key aspects of case-management may allow improved access to treatment for acutely malnourished children.

First, acute malnutrition is as a continuum condition, but it is arbitrarily divided into moderate (MAM) and SAM categories. This distinction results in separate programs overseen by different UN agencies and using

For peer review only - http://bmjopen.bmj.com/site/about/guidelines.xhtml

different protocols and products – ready to use supplementary food (RUSF) or fortified-blended flours for children with MAM and ready to use therapeutic food (RUTF) for children with SAM. This complicates supply chain, delivery of care, data collection, and creates confusion and extra work for caregivers and health workers alike (12). In practice, only SAM treatment is often available, whereas identification and treatment of children earlier in the wasting process would lead to fewer hospitalizations and deaths. The treatment of MAM has been shown to reduce mortality risk by more than 10% (13).

Second, current case definitions to determine program eligibility are un-necessarily complicated, using two independent anthropometric criteria: middle upper arm circumference (MUAC) or weight-for-height Z-score (WHZ). WHZ alone or in combination with MUAC does not offer a clear advantage for identifying children at near-term risk of death over MUAC alone (14). MUAC-only programming is expanding as evidence accumulates that weight gain and MUAC gain track each other and respond to treatment in similar ways (15–17). MUAC is therefore becoming a stand-alone practical tool for all phases of nutrition programming: screening, admission, monitoring recovery and determining discharge. Furthermore, the simplicity of MUAC bracelets makes it possible to screen children not only in the community, but also at home (18).

Third, therapeutic food rations could be allocated more efficiently. The RUTF ration for treating SAM (130-200 kcal/kg/day) is paradoxical. The amount of RUTF prescribed remains constant or even increases with weight gain up until discharge (i.e. as the child recovers from acute malnutrition), even though weight and MUAC gain is maximal during the first 2-3 weeks of supplementation (19). Consequently, gradual dose reduction seems to be a more rational use of RUTF, which accounts for a large proportion of total malnutrition programme costs (20).

Results on integrating SAM and MAM treatment in a single MUAC-based protocol using only RUTF at a gradually reduced dose were first published in 2015. This cluster-randomized trial conducted in Sierra Leone found high recovery rates, a reduced caseload of children with SAM, a better coverage rate, and reduced RUTF costs when compared to the standard protocol (21). More recently, a non-inferiority randomized trial in Burkina Faso showed that the reduction of the RUTF dose after the first two weeks results in similar weight gain velocity and recovery rates as with the standard dose given throughout SAM treatment (22).

Our research consortium developed the Optimizing treatment for Acute Malnutrition (OptiMA) strategy that simplifies the definition of acute malnutrition to MUAC < 125 mm or the presence of bipedal oedema, and

For peer review only - http://bmjopen.bmj.com/site/about/guidelines.xhtml

uses a single product for treatment – RUTF – at a gradually reduced dose based on a child's weight and MUAC status **(see Table 1).** The OptiMA strategy was first implemented in a proof-of-concept single-arm trial in Burkina Faso and showed a recovery rate that exceeded national and international SPHERE standards for both SAM and MAM (86.3%; 95%CI: 85.4-87.2) with excellent health worker adherence to the new RUTF dosage table (23). However, recovery of children admitted at MUAC <115 mm was poorer than anticipated (70.4%; 95 % CI 67.5-73.5), which necessitated a randomized controlled trial to compare OptiMA to current national malnutrition protocols. We therefore planned this individually randomized non-inferiority clinical trial to compare the OptiMA strategy to the DRC nutritional standard protocol in children 6-59 m with uncomplicated AM.

We hypothesized that the OptiMA strategy would be as effective as the current DRC national protocol currently in use, in children 6 to 59 years old as judged by the success rate in the treatment of uncomplicated severe and moderate acute malnutrition at 6 months post-randomization and in terms of recovery rate in the treatment of uncomplicated severe acute malnutrition.

Table 1: Wasting definition, treatment products, calculation of dosage and recovery definition in the DRC national and OptiMA protocol

	National DRC Protocol		OptiMA Protocol		
	**SAM**	**MAM**	**Acute malnutrition**		
**Wasting definition**	MUAC<115mm Or WHZ<-3 Or Bipedal oedema	MUAC [115mm-124] Or -3 < WHZ <-2	MUAC < 125mm Or Bipedal oedema		
**Treatment product**	RUTF 150-200 Kcal/kg/d	Super cereal plus 200 g/d (~1000Kcal/d) Or RUSF, one 92g sachet /d (500Kacl/d)	MUAC < 115 mm Or bipedal oedema	MUAC [115-119 mm]	MUAC [120-124 mm]
			RUTF 170-200 (Kcal/Kg/d)	RUTF 125-190 (Kcal/Kg/d)	RUTF 50-166 (Kcal/Kg/d)

For peer review only - http://bmjopen.bmj.com/site/about/guidelines.xhtml

**Calculation of dosage**	According to the weight	Fixed amount, regardless of weight or MUAC status	According to MUAC status and weight
**Recovery definition**	MUAC≥125mm Or WHZ ≥-1.5	MUAC≥125 mm Or WHZ ≥-1.5 If After recovery from SAM: MUAC≥125mm And WHZ ≥-1.5 And discharge after 3 months.	MUAC≥125mm for two consecutive weeks
	No oedema for two consecutive weeks and Minimum 4 weeks in program and good clinical health		No oedema for two consecutive weeks and Minimum 4 weeks in program and good clinical health

For peer review only

Objectives

The principal objective aims to determine, 6 months after inclusion, whether the OptiMA strategy leads a success rate that is non-inferior to that of the standard DRC protocol in use in the same outpatient health facilities. The definition of "success" is described in the Study Outcomes section.

The main secondary objective is to determine whether the recovery rate of children with uncomplicated SAM according to the current World Health Organization (WHO) definition (24) (i.e. MUAC <115 mm or WHZ<-3 or bilateral oedema) managed under the OptiMA protocol is non inferior to that of the national standard protocol, anticipating that such children treated under OptiMA will receive a smaller overall RUTF ration than those treated under the DRC national protocol.

Other secondary objectives include description and comparison between the two study arms of the following outcomes: RUTF consumption, relapse after nutritional recovery, outcomes compared to international SPHERE references, recovery and success in children who present with both wasting and stunting at inclusion, and to describe the nutritional and clinical status of children hospitalized while enrolled in the study.

Methods

This protocol follows the Standard Protocol Items: recommendations for Interventional Trials (SPIRIT)

For peer review only - http://bmjopen.bmj.com/site/about/guidelines.xhtml

guidelines (25).

Design

This study is a non-inferiority individually randomized-controlled clinical trial conducted at health centres and in the community. Participants were randomly assigned to either the OptiMA strategy arm (intervention = OptiMA) or the national standard protocol arm (control = Standard).

Study Setting

The trial took place in the Kamuesha health zone, Kasai province in DRC and was nested within a medical and nutritional emergency humanitarian project launched by ALIMA in May 2018. The Kamuesha health zone is located in a tropical forest and is particularly isolated.

ALIMA supports the DRC Ministry of Health (MoH) in 9 of 26 health areas in the Kamuesha health zone to address a nutritional crisis occurring after a two years of armed conflict with significant population displacement (26). During the trial preparation phase, we visited all health areas covered by ALIMA and selected four as research sites based on demographic, epidemiologic and logistical criteria in addition to observations provided by local stakeholders. According to projections from the last census, the four health areas chosen covered a population of 65,000 people, including 12,000 children aged 6 to 59 months, spread over 60 villages. Health facilities included in the study were the four community-based primary health centres of these four health areas in addition to the general hospital of Kamuesha district, which was used for referring participants with medical complications. Most (N=49) villages were less than 10 kilometres away from the primary health centres while 11 were more than 15 kilometres away.

Eligibility criteria

Children 6–59 m, residing in one of the four health areas and meeting at least one of the three following AM criteria were eligible for inclusion in the trial: MUAC <125 mm and/or Bilateral pitting oedema (+/++) and/or WHZ<-3. Children with a medical complication requiring hospitalization, no appetite, oedema grade +++, with a known allergy to milk, peanuts or RUTFs, with a known chronic pathology (such as sickle cell anaemia, trisomy 21, congenital heart disease, neurological disease), or already enrolled in a malnutrition programme were excluded from the trial. Two categories of children were included in the trial but not eligible for randomization for ethical concerns. First, children with WHZ<-3 and MUAC $\geq$125mm without oedema were systematically included in the control arm, as they were not RUTF eligible according to the wasting definition of the OptiMA strategy. Second, children with a sibling already enrolled in the study were systematically allocated to the study arm of the index sibling.

For peer review only - http://bmjopen.bmj.com/site/about/guidelines.xhtml

Study outcomes

The primary outcome will be judged by a binary composite indicator. Children classified as “success” fulfil all of the following criteria: alive, not acutely malnourished per the definition applied at inclusion, and not having an additional episode of acute malnutrition throughout the 6-month observation period. All other children are classified as “unsuccessful”.

The main secondary outcome will be determined among children in both arms of the trial who meet the current WHO definition of SAM at inclusion. For this sub-group, recovery is defined after a 4-week minimum duration of treatment as clinically well, absence of bipedal oedema, and a MUAC $\geq$ 125 mm for the OptiMA arm or MUAC $\geq$125 mm or WHZ $\geq$-1.5Z for the standard arm. Additional secondary outcomes are listed in **Table 2**.

Table 2: Outcomes

	Measurement variable	Denominator	Method of aggregation	Time point
**Primary**				
Success	Composite : alive, not acutely malnourished per the definition applied at inclusion and no additional episode of acute malnutrition throughout the 6- month observation period	Children with MUAC < 125 or WHZ <-3 or bipedal oedema at inclusion	Proportion	6 months post-inclusion
**Main secondary**				
Recovery	Composite: a 4 week minimum duration of RUTF treatment, absence of bipedal oedema and for the OptiMA arm a MUAC $\geq$ 125 mm and for the standard arm MUAC $\geq$ 125 mm or WHZ $\geq$-1.5	Children with MUAC < 115 or WHZ <-3 or bipedal oedema at inclusion	Proportion	End of RUTF treatment initiated at inclusion
**Secondary**				
Consumption of RUTF per children in success	Sachets of RUTF consumed	Children with MUAC < 125 or WHZ <-3 or bipedal oedema at inclusion	Mean	6 months post-inclusion
Consumption of RUTF per SAM children at	Sachets of RUTF consumed	Children with MUAC < 115 or WHZ <-3 or	Mean	End of RUTF treatment

For peer review only - http://bmjopen.bmj.com/site/about/guidelines.xhtml

inclusion recovered		bipedal oedema at inclusion		initiated at inclusion
Average number of children in success for an equivalent amount of RUTF	Children cured with 1500 sachets of RUTF	Children with MUAC < 125 or WHZ <-3 or bipedal oedema at inclusion	Mean	6 months post-inclusion
Average number of MAS children at inclusion cured for an equivalent amount of RUTF	Children cured with 1500 sachets of RUTF	Children with MUAC < 115 or WHZ <-3 or bipedal oedema at inclusion	Mean	End of RUTF treatment initiated at inclusion
Relapse to SAM WHO definition	Children with MUAC<115 or WHZ <-3 or oedema after RUTF treatment	Children with MUAC < 115 or WHZ <-3 or bipedal oedema at inclusion	Proportion	3 months following recovery from SAM episode at inclusion
Average daily weight gain in SAM children recovered	g/kg/day	Children with MUAC < 115 or WHZ <-3 or bipedal oedema at inclusion	Mean	End of RUTF treatment initiated at inclusion
Average daily MUAC gain in SAM children recovered	mm/day	Children with MUAC < 115 or WHZ <-3 or bipedal oedema at inclusion	mean	End of RUTF treatment initiated at inclusion

Sample size considerations

We determined the sample size for the principal objective using an expected success rate of 55% and for the secondary main objective an expected recovery rate of 85%. Both of these hypotheses are based on recovery, death, default and relapse rates available in the literature for severely and moderately wasted children supplemented with RUTF and/or RUSF in DRC and other African contexts (27,28). We also considered spontaneous recovery rate documented for MAM children without treatment in the standard arm. (29). During the preparation phase, we twice visited the four sites included in the study and found that the standard MAM treatment program was either non- or partially -functional.

We assumed a margin of non-inferiority of 10%, the same as the three other non-inferiority trials assessing simplified treatment strategies with RUTF for wasted children (30–32).

For peer review only - http://bmjopen.bmj.com/site/about/guidelines.xhtml

To demonstrate non-inferiority of the OptiMA strategy compared to standard protocol, 890 and 490 participants were required for the principal and main secondary objectives, respectively, with 80% power at a unilateral significance level of 2.5% and with an inflation of 15% to account for un-exploitable data. We estimated 15% of incomplete data in the light of the challenging reality of this study setting where families are regularly confronted with barriers to receiving healthcare, insecurity, and displacement.

Randomization

The trial statistician established and maintained a confidential randomization list. Randomization was done in blocks, and was stratified at the study site by the severity of acute malnutrition according to the WHO definition. This double stratification allows for recruitment of comparable groups of SAM and non-SAM children and for simultaneous randomization at the four sites. The trial data scientist integrated the list into a randomization software developed for the trial that was programmed on four tablet devices. This software assigned a treatment arm by sequentially drawing from this list each time a randomization procedure was completed. Once the sample size for the primary objective was reached, only WHO-SAM cases continued to be enrolled and randomized until the sample size required for the main secondary objective was attained.

Patient and Public Involvement

To ensure that the trial was understood, accepted, and implemented in accordance with local customs and practices, meetings to explain trial eligibility and procedures were organized with community representatives in each village and with nutrition actors prior to recruitment. During the trial's active phase, regular oral presentations were held at the local level. We intend to disseminate the findings through meetings with community representatives and national nutrition actors.

Enrolment and informed consent procedure

Prior to enrolment, all community health workers, nurse research officers, and Ministry of Health medical staff implicated in the study were trained on the trial protocol, standardized operating procedures, and the correct use of tools developed for data collection. Nurse research officers were specifically trained to enrol and randomize study participants, and monitor home visits and outpatient visits at the health centre. Children were recruited through monthly active mass malnutrition screenings in each of the 60 villages. Concomitantly, children attending outpatient consultations at the four study health centres underwent routine passive malnutrition screening and were enrolled in the trial if eligible. In both active and passive screening, a nurse research officer, assisted by a community health worker, collected anthropometric (weight, height, MUAC, WHZ) and clinical (presence of oedema) information on eligible children. Children then underwent a medical consultation to determine whether they met the criteria for study inclusion. A

For peer review only - http://bmjopen.bmj.com/site/about/guidelines.xhtml

standard operating procedure **(see additional files 1)** for assessing child anthropometry (weight, height, MUAC and oedema) were observed in each site. For each eligible child, the caretaker was given a study information sheet detailing the study's aims, treatment protocols, study duration, and frequency of clinic visits and community follow-up. This information was explained orally in Tshiluba (local language). Caretakers who agreed to participate in the study indicated their consent by signing (signature or fingerprint) a written consent form. When caretakers were not able to read or write, an impartial witness oversaw the consent process and attested to the caretaker's consent by signing the consent form on her/his behalf. All caretakers were informed about their right to withdraw from the study at any time without affecting the quality of medical care provided to their children. All medical care and nutritional treatment were provided free of charge, regardless of participation in the study.

Intervention and follow-up

In the intervention arm, all children with a MUAC<125mm or oedema were supplemented with RUTF, according to the OptiMA dosage table based on the progression of MUAC and weight gain during recovery (i.e. RUTF dosage is gradually reduced as a child's weight and MUAC increases) **(see Table 1).** The OptiMA RUTF dosage table provides for a daily caloric intake of 170-200, 125-190 and of 50-166 Kcal/Kg/day in children with a MUAC <115 mm, between 115 and 119 mm and >119 mm, respectively. Children with oedema received the same RUTF ration as children with MUAC <115 mm until oedema resolved, at which point their ration was determined by MUAC and weight **(see additional files 2).**

In the control arm, children included with MUAC <115 mm and/or WHZ<-3 and/or nutritional oedema, were treated with RUTF, according to the DRC national protocol dosage table which is based on a child's weight at each visit (i.e. RUTF dosage gradually increases as a child's weight increases). The DRC protocol provides a RUTF daily caloric intake of 150-200 Kcal/kg/day during the entire treatment course until recovery in children with SAM and one sachet of RUSF per day (500Kcal/day) in children with MAM.

The differences between the OptiMA and the DRC national standard protocols, summarized in **Table 1**, concern treatment eligibility, definitions of recovery and relapse, the type of therapeutic food provided, and RUTF dosage calculation. All other aspects of the standard nutrition protocol were applied to all children in both arms. All cases of SAM according to the WHO definition received Amoxicillin 50-100 mg/kg/day for 7 days, and all children received vitamin A and deworming regardless of anthropometry. In both arms, a malaria rapid test was systematically performed at inclusion and at any point during follow-up when a child presented with clinical signs of malaria. If positive, an Artemisinin-combination treatment was prescribed

For peer review only

For peer review only - http://bmjopen.bmj.com/site/about/guidelines.xhtml

for the child. At the time of the study, the HIV national program was not functional in the Kamuesha health zone, hence children with SAM were not offered HIV counselling and testing, as is recommended (24).

All participants were monitored for 6 months after inclusion **(see Figure 1).** While in the treatment phase, children came to the health centres once a week for outpatient consultations (or every two weeks for those children living in villages more than 14 km from the health centre). Upon discharge from treatment, or immediately after inclusion for children with MAM who did not receive RUTF in the standard arm, children were consulted every two weeks in their villages until 6 months post-inclusion was completed. During village visits, a nurse research officer assisted by one or two community health workers monitored the anthropometric and clinical status of these children, referring any child who needed nutritional or medical care to either the primary health care facility or to the Kamuesha general hospital.

Blinding

This was an un-blinded trial. Caretakers and study personnel were aware of study arm allocation for participating children due to differences in anthropometric criteria and RUTF ration. Upon inclusion, the nurse research officer gave each trial participant's caretaker a card specifying the nutritional strategy being followed by the child. Caretakers were asked to present this card in the event of an unplanned outpatient consultation or hospital admission during the study period. In this way, nurses or doctors at any health facility were able to identify the nutritional treatment being followed by the child.

Data collection and management

After obtaining individual informed consent, all socio-demographic characteristics, anthropometric and clinical data collected during follow up were documented in the case report forms by the research officers and the health care providers trained specifically for this study. All data entered into the database were securely transferred and stored on a server hosted by PAC-CI, a satellite for the French National Agency for Research on AIDS and Viral Hepatitis in Abidjan, Côte d'Ivoire, which served as the methodology and coordinating centre for this study. All data and any adverse events, including deaths and hospitalisations, were rigorously monitored both on-site and remotely, according to the data monitoring plan. To ensure patient safety and data integrity, the methodology and coordinating centre continuously supervised data management activities, and the senior scientific project leader and investigators performed field visits on a regular basis. If children failed to attend follow-up visits, the community health worker living in the village visited the household to check on the child, determine the reasons for absence and to encourage the caretakers to adhere to the planned health centre visit schedule.

For peer review only - http://bmjopen.bmj.com/site/about/guidelines.xhtml

Statistical analyses

All statistical analyses will be performed using RStudio software (Inc., Boston, Massachusetts, USA) as specified by the data analysis plan published on clinicaltrials.gov prior to performing any analysis. Statistical tests will be carried out bilaterally with an alpha risk of 5%, and unilaterally with an alpha risk of 2, 5% for the non-inferiority analysis. Qualitative variables will be described in terms of numbers, percentages, and provided with confidence intervals when relevant. If necessary, comparisons of qualitative variables will be made using Chi-squared or Fisher exact tests. Quantitative variables will be described in terms of mean, standard deviation and confidence interval or median, range and interquartile range. If necessary, comparisons of quantitative variables will be made using Student's, Wilcoxon's or the Kruskal-Wallis tests, depending on the distribution of the variable of interest. Time delay type variables will be described in terms of the incidence of occurrence, and the probability of occurrence over time, estimated by the Kaplan-Meier method. If necessary, probability comparisons will be made using log-rank tests, or proportional risk models, after verification of the assumption of proportionality of risks.

Primary analysis of the primary and secondary main outcomes

Descriptive summaries of participant characteristics by arm at inclusion will be tabulated. Participants included in the main analyses will be described according to the diagram defined by CONSORT recommendations (33). The occurrence of the primary "success" and the secondary main outcome "recovery" will be compared between the two randomization arms. These two comparisons will be performed both by "intention to treat" basis and on a "per-protocol" basis. Non-inferiority analyses will be performed on these two outcomes. Only if non-inferiority for these two outcomes is demonstrated, will the other secondary analyses will be performed, and if appropriate, as a superiority analysis. Superiority will be particularly sought for cost outcomes related to RUTF cost-efficiency.

Sensitivity analysis of the primary outcome

First, rates of "success" and "recovery" will be analysed based on all available data. Then a sensitivity analysis will be performed using the maximum bias method. In this analysis, deceased participants, those who have withdrawn their consent, who have been transferred to another health facility or have been lost to follow-up, will be considered to have systematically failed to the allocated treatment.

Safety

For peer review only - http://bmjopen.bmj.com/site/about/guidelines.xhtml

The trial was monitored monthly by an international steering committee and quarterly by a national steering committee where any adverse events were presented. No interim analysis was planned before the final analyses. The DSMB could have requested an interim analysis if it deemed it necessary for patient safety.

A child in intervention arm could be removed from the study for two reasons: 1. if he or she had two episodes of >5% weight loss during the course of treatment; and 2. if he or she was not SAM at inclusion but deteriorated to SAM during RUTF supplementation.

Ethics and dissemination

We obtained ethical approval with annual renewal from DRC National Ethics Committee (CNES) and from the Ethics Evaluation Committee of the French National Institute for Health and Medical Research (INSERM). In November 2019, the CNES performed an audit at each site. All data were anonymized when entered into the database using unique identification numbers. We intend to disseminate the results regardless of positive or negative findings broadly via peer-reviewed publication, conferences, and clinical networks targeting academics, policy- makers, clinicians and caregivers.

Discussion

This trial was anchored in the following foundational principle: the need to merge MAM and SAM nutritional care into a single program using one anthropometric admission criterion and one type of therapeutic food in order to simplify care for families, health workers, and program managers. This study will contribute to a developing evidence-base composed of similar studies already published or currently in progress in Sierra Leone, Burkina Faso, South Sudan and Kenya (30,34) . Given that the risk-profile associated with low anthropometry is highly contextual, it is important to conduct similar trials in multiple settings. This will be the first such trial in DRC, where we expect a higher proportion of oedematous malnutrition than is typically found in East or West Africa. Furthermore, this is an individually randomized trial that will monitor outcomes for an extended period, 4-5 months, after nutritional recovery. It also includes a group of children rarely studied but common in everyday practice: MAM children who do not receive supplemental food. This study design should allow for robust conclusions not only regarding the non-inferiority of OptiMA compared to a typical standard SAM protocol, but also an evaluation of the incidence of SAM among children with MAM. This trial will also document outcomes of sustained health and nutrition status over a longer period than previous trials.

Furthermore, the stratified randomisation of children with SAM according to the WHO definition will shed some light on the clinical effects of the OptiMA strategy for this specific category of children. This inclusion

For peer review only - http://bmjopen.bmj.com/site/about/guidelines.xhtml

strategy will also help to determine the segment of children with SAM defined by only WHZ with MUAC >125 mm, in Kasaï province where stunting prevalence in children is higher than anywhere else in the DRC (35).

Trial status

The recruitment phase began in July 2019 and ended 22 January 2020. The estimated completion date for this study is September 2020.

Abbreviations

ALIMA: The Alliance for International Medical Action ; AM: acute malnutrition; CNES: Comité National d'Ethique de la Santé); DRC: Democratic Republic of Congo; DSMB: data safety monitoring board; MAM: moderate acute malnutrition; MoH: Ministry of Health; MUAC: Mid Upper Arm Circumference; OptiMA: Optimizing treatment for Acute Malnutrition; RUSF: ready to use therapeutic food ; RUTF: ready-to-use therapeutic food; SAM: severe acute malnutrition; SD: standard deviation; UN: united nations ; WHO: World Health Organization; WHZ: weight-for-height Z-score.

Declarations

Ethics approval

The study was conducted in accordance with the Declaration of Helsinki. The protocol was approved by the DRC national ethics committee (CNES) (94/CNES/BN/PMMF/2018) and by the Ethics Evaluation Committee of Inserm, the French National Institute for Health and Medical Research (Comité d'Evaluation Ethique de l'Inserm) (18-545). The final version of the protocol, version 3.1, dated 22 October 2019, is available for sharing on request, and included three minor amendments to protocol version 2.0 (dated 6 March 2019) and version 3.0 (dated 18 April 2019), which was shared with national and international ethical committees and did not require a new authorization. In November 2019, the CNES performed a field visit at each of the study sites and then renewed approval (152/CNES/BN/PMMF/2019) for the continuation of the trial.

Consent for publication

Not required.

Data statement and sharing

A data sharing plan is available in the corresponding registry record. In accordance with the International Committee of Medical Journal Editors (ICMJE), the data generated by the trial will be made available after 6 months and ending 36 months following article publication. After, the data will be available in our University's data warehouse and in co-investigators (Programme National Nutrition of DRC) data warehouse

For peer review only - http://bmjopen.bmj.com/site/about/guidelines.xhtml

upon request from researchers who provide a methodologically sound proposal and whose proposed use of the data has been approved by an independent review committee.

Competing interests

KP serves on the Social Purposes Advisory Commission of Nutriset, a main producer of lipid-based nutrient supplement products. No other interests are declared.

Funding statement

The study was supported by the Innocent Foundation (London, UK), grant number ALIMA-2018-DRC, and received additional funding from European Commission through the European Civil Protection and Humanitarian Aid Operations (ECHO, Brussels, Belgium), grant number ECHO/COD/BUD/2018/91023. The funders had no role in study design, data collection and analysis, decision to publish, or preparation of the manuscript.

This document covers humanitarian aid activities implemented with the financial assistance of the European Union. The views expressed herein should not be taken, in any way, to reflect the official opinion of the European Union, and the European Commission is not responsible for any use that can be made of the information it contains.

Authors contributions

SS and RB developed the clinical (SS) and methodological (RB) study concept. RB, CC, XA, DG, AA, KP and SS designed the study methodology and wrote the protocol. CC, VH, HB, RA and MK coordinate the study teams. LIB, GTS coordinate the Ministry of Health staff working in the trial. CC, VH, HB, LIB, GTS, AB, CY and RB organize and supervise data collection. CY created the software tool for randomization and developed the database. CC, VH, DG, XA, SS and RB developed the statistical analysis strategy. CC wrote the first draft of the manuscript. SS and RB were primarily responsible for the final content of the manuscript. All authors critically reviewed the first draft and had a substantial writing contribution to the development of the final manuscript.

Acknowledgements

We are indebted to the women and children who participate in the study. The authors wish to particularly acknowledge the ALIMA operational team on the ground who managed the study and the nutrition program on a day-to-day basis. The authors also acknowledge the Ministry of Health of the Democratic Republic of Congo through the commitment of key representatives from the Kamuesha health zone and the National Nutrition Programme. We acknowledge the implementing partners of the inter-agency Nutrition Cluster Kasaï Province, in particular representatives from UNICEF and the World Food Programme. We also

For peer review only - http://bmjopen.bmj.com/site/about/guidelines.xhtml

acknowledge Maguy Daures (Inserm UMR 1219, Bordeaux), Valérie Journot (Inserm UMR 1219, Bordeaux) and Claire Levy-Marchal (ALIMA, Paris) for having advising the study team on the trial methodology.

This study was developed as part of the Clinical and Operational Research Alliance (CORAL). This research platform aims at developing high-quality innovative and transformative research programs within a partnership between scientists from the research institute Inserm (Institut National de la Santé et la Recherche Médicale, Bordeaux and Abidjan) and the humanitarian organization ALIMA (Alliance for International Medical Action), primarily on improving maternal and child health outcomes. A board of directors defines the scientific policy of the CORAL partnership alongside with the supervision of research projects and dissemination of results. It consists of senior representatives from both ALIMA and Inserm: Renaud Becquet (Inserm Bordeaux, methodological co-chair), Susan Shepherd (ALIMA, medical co-chair), Augustin Augier (ALIMA), Moumouni Kinda (ALIMA), Marie Jaspard (ALIMA & Inserm Abidjan), Claire Levy-Marchal (ALIMA), and Xavier Anglaret (Inserm Bordeaux & Abidjan). The CORAL research platform meets annually with an external scientific advisory board to review projects and strategic orientation.

Finally, we warmly acknowledge the members of the Data Safety Monitoring Board (DSMB) for the OptiMA-DRC trial: Yves Martin-Prevel (DSMB chair, French National Institute for Sustainable Development (IRD), NUTRIPASS team, University of Montpellier, Montpellier, France), Matthew Colderon (Epicentre, New York, USA) and Katia Castetbon (DSMB statistician, School of Public Health, Université Libre de Bruxelles, Bruxelles, Belgium).

References

1. Levels and trends in child malnutritionUNICEF / WHO / World Bank Group Joint Child Malnutrition EstimatesKey findings of the 2019 edition [Internet]. [cité 18 nov 2019]. Disponible sur: https://www.who.int/nutgrowthdb/jme-2019-key-findings.pdf?ua=1

2. Black RE, Victora CG, Walker SP, Bhutta ZA, Christian P, de Onis M, et al. Maternal and child undernutrition and overweight in low-income and middle-income countries. The Lancet. 3 août 2013;382(9890):427‑51.

3. Isanaka S, Boundy EO, Grais RF, Myatt M, Briend A. Improving Estimates of Numbers of Children With Severe Acute Malnutrition Using Cohort and Survey Data. Am J Epidemiol. 15 déc 2016;184(12):861‑9.

4. Hure A, Oldmeadow C, Attia J. Invited Commentary: Improving Estimates of Severe Acute Malnutrition Requires More Data. Am J Epidemiol. 15 déc 2016;184(12):870‑2.

For peer review only - http://bmjopen.bmj.com/site/about/guidelines.xhtml

5. Unicef. UNICEF DRC Humanitarian Situation Report March, 2018 - Democratic Republic of the Congo [Internet]. ReliefWeb. [cité 16 janv 2020]. Disponible sur: https://reliefweb.int/report/democratic-republic-congo/unicef-drc-humanitarian-situation-report-march-2018

6. WHO child growth standards and the identification of severe acute malnutrition in infants and children - A Joint Statement by the World Health Organization and the United Nations Children's Fund- 2009 [Internet]. [cité 16 janv 2020]. Disponible sur: https://apps.who.int/iris/bitstream/handle/10665/44129/9789241598163_eng.pdf;jsessionid=7C1A602ED39C4B7995CBE93E690CA58C?sequence=1

7. World Health Organization, Nutrition for Health and Development. Guideline. [Internet]. 2013 [cité 16 janv 2020]. Disponible sur: http://www.ncbi.nlm.nih.gov/books/NBK190328/

8. Collins S, Sadler K, Dent N, Khara T, Guerrero S, Myatt M, et al. Key Issues in the Success of Community-Based Management of Severe Malnutrition. Food Nutr Bull. sept 2006;27(3_suppl3):S49‑82.

9. No Wasted Lives Brochure 2018 [Internet]. [cité 18 nov 2019]. Disponible sur: https://static1.squarespace.com/static/58da81cdd1758e39ca705526/t/5a7adf8171c10b8aa3b4462f/1518002053092/No+Wasted+Lives+Brochure+2018.pdf

10. Coalition No Wasted Lives. Etat de la malnutrition [Internet]. État de la malnutrition aiguë. [cité 13 janv 2020]. Disponible sur: https://acutemalnutrition.org/fr/countries

11. Horton, Susan, Shekar, Meera, McDonald, Christine, Mahal, Ajay, Krystene Brooks, Jana. Scaling Up Nutrition : What Will it Cost? [Internet]. The International Bank for Reconstruction and Development / The World Bank; 2010. Disponible sur: http://hdl.handle.net/10986/2685

12. Rogers E, Myatt M, Woodhead S, Guerrero S, Alvarez JL. Coverage of community-based management of severe acute malnutrition programmes in twenty-one countries, 2012-2013. PloS One. 2015;10(6):e0128666.

13. Isanaka S, Menzies NA, Sayyad J, Ayoola M, Grais RF, Doyon S. Cost analysis of the treatment of severe acute malnutrition in West Africa. Matern Child Nutr [Internet]. oct 2017 [cité 18 nov 2019];13(4). Disponible sur: https://onlinelibrary.wiley.com/doi/abs/10.1111/mcn.12398

14. Briend A, Maire B, Fontaine O, Garenne M. Mid-upper arm circumference and weight-for-height to identify high-risk malnourished under-five children. Matern Child Nutr. janv 2012;8(1):130‑3.

15. Binns P, Dale N, Hoq M, Banda C, Myatt M. Relationship between mid upper arm circumference and weight changes in children aged 6–59 months. Arch Public Health. déc 2015;73(1):1‑10.

16. Roberfroid D, Hammami N, Lachat C, Weise Prinzo Z, Sibson V, Guesdon B, et al. Utilization of mid-upper arm circumference versus weight-for-height in nutritional rehabilitation programmes: a systematic review of evidence. 2013 [cité 13 janv 2020]; Disponible sur: http://hdl.handle.net/1854/LU-5700355

For peer review only - http://bmjopen.bmj.com/site/about/guidelines.xhtml

17. Burza S, Mahajan R, Marino E, Sunyoto T, Shandilya C, Tabrez M, et al. Community-based management of severe acute malnutrition in India: new evidence from Bihar. Am J Clin Nutr. avr 2015;101(4):847‑59.

18. Alé FGB, Phelan KPQ, Issa H, Defourny I, Le Duc G, Harczi G, et al. Mothers screening for malnutrition by mid-upper arm circumference is non-inferior to community health workers: results from a large-scale pragmatic trial in rural Niger. Arch Public Health [Internet]. déc 2016 [cité 18 nov 2019];74(1). Disponible sur: http://archpublichealth.biomedcentral.com/articles/10.1186/s13690-016-0149-5

19. Goossens S, Bekele Y, Yun O, Harczi G, Ouannes M, Shepherd S. Mid-upper arm circumference based nutrition programming: evidence for a new approach in regions with high burden of acute malnutrition. PloS One. 2012;7(11):e49320.

20. Shekar M, Kakietek J, Dayton Eberwein J, Walters D. An Investment Framework for Nutrition: Reaching the Global Targets for Stunting, Anemia, Breastfeeding, and Wasting [Internet]. The World Bank; 2017 [cité 21 nov 2019]. Disponible sur: http://elibrary.worldbank.org/doi/book/10.1596/978-1-4648-1010-7

21. Maust A, Koroma AS, Abla C, Molokwu N, Ryan KN, Singh L, et al. Severe and Moderate Acute Malnutrition Can Be Successfully Managed with an Integrated Protocol in Sierra Leone. J Nutr. 1 nov 2015;145(11):2604‑9.

22. Kangas ST, Salpéteur C, Nikièma V, Talley L, Ritz C, Friis H, et al. Impact of reduced dose of ready-to-use therapeutic foods in children with uncomplicated severe acute malnutrition: A randomised non-inferiority trial in Burkina Faso. Persson LÅ, éditeur. PLOS Med. 27 août 2019;16(8):e1002887.

23. Daures M, Phelan K, Issoufou M, Kouanda S, Sawadogo O, Issaley K, et al. New approach to simplifying and optimizing acute malnutrition treatment in children aged 6 to 59 months: the OptiMA single-arm proof-of-concept trial in Burkina Faso. Br J Nutr. 10 déc 2019;1‑31.

24. Community Based Management of Sever Acute Malnutrition - A Joint Statement by the World Health Organization, the World Food Programme, the United Nations System Standing Committee on Nutrition and the United Nations Children's Fund - 2007 [Internet]. [cité 18 nov 2019]. Disponible sur: https://www.unicef.org/publications/files/Community_Based_Management_of_Sever_Acute__Malnutirtion.pdf

25. Chan A-W, Tetzlaff JM, Altman DG, Laupacis A, Gøtzsche PC, Krleža-Jerić K, et al. SPIRIT 2013 statement: defining standard protocol items for clinical trials. Ann Intern Med. 5 févr 2013;158(3):200‑7.

26. DR Congo - Situation Report N°14 : Complex Emergency in the Kasai Region (23 octobre 2017) | HumanitarianResponse [Internet]. [cité 20 mars 2020]. Disponible sur: https://www.humanitarianresponse.info/en/operations/democratic-republic-congo/document/dr-congo-situation-report-n%C2%B014-complex-emergency-kasai

27. O'Sullivan NP, Lelijveld N, Rutishauser-Perera A, Kerac M, James P. Follow-up between 6 and 24 months after discharge from treatment for severe acute malnutrition in children aged 6-59 months: A systematic review. PLOS ONE. 30 août 2018;13(8):e0202053.

For peer review only

For peer review only - http://bmjopen.bmj.com/site/about/guidelines.xhtml

28. Grellety E, Babakazo P, Bangana A, Mwamba G, Lezama I, Zagre NM, et al. Effects of unconditional cash transfers on the outcome of treatment for severe acute malnutrition (SAM): a cluster-randomised trial in the Democratic Republic of the Congo. BMC Med. 26 2017;15(1):87.

29. James P, Sadler K, Wondafrash M, Argaw A, Luo H, Geleta B, et al. Children with Moderate Acute Malnutrition with No Access to Supplementary Feeding Programmes Experience High Rates of Deterioration and No Improvement: Results from a Prospective Cohort Study in Rural Ethiopia. PloS One. 2016;11(4):e0153530.

30. Bailey J, Lelijveld N, Marron B, Onyoo P, Ho LS, Manary M, et al. Combined Protocol for Acute Malnutrition Study (ComPAS) in rural South Sudan and urban Kenya: study protocol for a randomized controlled trial. Trials [Internet]. déc 2018 [cité 18 nov 2019];19(1). Disponible sur: https://trialsjournal.biomedcentral.com/articles/10.1186/s13063-018-2643-2

31. Bahwere P, Balaluka B, Wells JCK, Mbiribindi CN, Sadler K, Akomo P, et al. Cereals and pulse-based ready-to-use therapeutic food as an alternative to the standard milk- and peanut paste-based formulation for treating severe acute malnutrition: a noninferiority, individually randomized controlled efficacy clinical trial. Am J Clin Nutr. avr 2016;103(4):1145‑61.

32. Bahwere P, Banda T, Sadler K, Nyirenda G, Owino V, Shaba B, et al. Effectiveness of milk whey protein-based ready-to-use therapeutic food in treatment of severe acute malnutrition in Malawian under-5 children: a randomised, double-blind, controlled non-inferiority clinical trial. Matern Child Nutr. juill 2014;10(3):436‑51.

33. Moher D, Hopewell S, Schulz KF, Montori V, Gøtzsche PC, Devereaux PJ, et al. CONSORT 2010 Explanation and Elaboration: updated guidelines for reporting parallel group randomised trials. BMJ [Internet]. 24 mars 2010 [cité 17 janv 2020];340. Disponible sur: https://www.bmj.com/content/340/bmj.c869

34. Lelijveld N, Bailey J, Mayberry A, Trenouth L, N'Diaye DS, Haghparast-Bidgoli H, et al. The « ComPAS Trial » combined treatment model for acute malnutrition: study protocol for the economic evaluation. Trials. 24 avr 2018;19(1):252.

35. Kismul H, Acharya P, Mapatano MA, Hatløy A. Determinants of childhood stunting in the Democratic Republic of Congo: further analysis of Demographic and Health Survey 2013–14. BMC Public Health. 1 août 2017;18(1):74.

Figure 1: Schedule of enrolment, interventions and assessments OptiMA-DRC overview

d=day; m=month
*Monthly active screening in 60 villages and passive screening during outpatient visit in 4 health centre
**Weekly (for those living in villages at 14 km or less from the health centre) or bimonthly (for those living in villages more than 14 km from the health centre) outpatient visits at health centre for participants with RUTF supplementation
*** Bimonthly home visits for children without RUTF supplementation.

For peer review only - http://bmjopen.bmj.com/site/about/guidelines.xhtml

Figure 1: Schedule of enrolment, interventions and assessments OptiMA-DRC overview

		STUDY PERIOD							
	**Enrolment**	**Allocation**	**Post-allocation**						**Close-out**
**TIMEPOINT**		**d0**	m_1	m_2	m_3	m_4	m_5	m_6	m_6
**ENROLMENT:**									
**Eligibility screening***	X								
		Outpatient visit							
**Informed consent**		X							
**Allocation**		X							
**INTERVENTIONS:**		X	Outpatient or home visits **						
**Standard strategy**		X	X	X	X	X	X	X	
**OptiMA strategy**		X	X	X	X	X	X	X	
**ASSESSMENTS:**									
**Clinical examination**		X	X	X	X	X	X	X	X
**Anthropometry measurements**	X	X	X	X	X	X	X	X	X
			Inpatient wards						
**Hospitalization if required**			X	X	X	X	X	X	X

For peer review only

d=day; m=month

*Monthly active screening in 60 villages and passive screening during outpatient visit in 4 health centre

**Weekly (for those living in villages at 14 km or less from the health centre) or bimonthly (for those living in villages more than 14 km from the health centre) outpatient visits at health centre for participants with RUTF supplementation

*** Bimonthly home visits for children without RUTF supplementation.

Additional file 1. Standard operating procedure for taking anthropometric measurements

OptiMA study – The Democratic Republic of Congo

<table>
<tr><td rowspan="2">ALIMA</td><td rowspan="2">République Démocratique du Congo
Ministère de la Santé
Inserm</td><td colspan="2">SOP_01_OptiMA-RDC</td></tr>
<tr></tr>
<tr><td rowspan="2"></td><td rowspan="2">Procedure for taking anthropometric measurements</td><td colspan="2">Version</td></tr>
<tr><td>V1.0</td><td>24/05/2019</td></tr>
</table>

<table>
<tr><td colspan="2">Version</td><td>Modification</td></tr>
<tr><td>1.0</td><td>24/05/2019</td><td>Creation</td></tr>
</table>

For peer review only

Objective

This document is intended to describe the process of taking anthropometric measurements in the OptiMA -DRC clinical trial.

Scope of application

These procedures are intended to be used by the research team of the OptiMA-DRC trial, and more specifically for the nurses and community health workers to follow during the study.

Acronyms

AM: Acute Malnutrition

CHW: Community Health Workers

CMAM: Community Management of Acute Malnutrition

CTT: Clinical Trial Technician

IT: Titular Nurse of health centre

MUAC: Mid Upper Arm Circumference

WHZ: Weight for Height Z score

1. General principles

From the start of the inclusion phase of the trial, research teams will organize monthly active screening for acute malnutrition (AM) in villages in the study health zones. During these sessions, the community health workers (CHWs) previously trained for this purpose will take anthropometric measurements (weight, height, mid upper arm circumference (MUAC)) and check for bilateral nutritional oedema under the supervision of a nurse among all children aged 6 to 59 months present on the screening day and accompanied by a legal representative.

Children coming for a paediatric consultation at the health centre will also be screened for acute malnutrition by health centre staff using this procedure.

Anthropometric measurements will also be taken in the children included in the study during weekly follow-up visits to the health centre and during biweekly follow-up home visits.

2. Logistics/data collection

The equipment needed to take anthropometric measurements are:

- 25 kg Salter-type scale graduated to the nearest hundredth gram;
- A standard 5 kg weight;
- Height board;
- MUAC bracelets;
- Basins for weighing and soaps for cleaning the basins;
- Registry dedicated to active screening for AM in villages as part of the OptiMA-DRC trial;
- Dedicated registry for passive AM screening in health centres as part of the OptiMA-DRC trial;
- Therapeutic follow-up sheet used for children in the study at the inclusion visit.

3. Organization of activities

Concerning active screening in the villages and home follow-up visits, the nurse will travel by motorbike to the villages in his or her health zone according to the planned tours. He will bring a Salter scale, a 5 kg weight, a height board, the register for active screening or the therapeutic sheets for home follow-up visits.

The basins will be placed in each health area by axis, according to the geographical position and distance between villages. After each daily activity, the CHWs will proceed to clean the basin.

At the level of the health centre, the health and research staff will ensure that the equipment is available and functional on a daily basis.

4. Techniques

<u>Weight:</u>

Tare the scale before weighing the 5 kg weight. Repeat the tare on the scale every 10 children weighed.

To check the accuracy of the scale:

- Put the standard 5kg weight on the scale;
- If the scale does not read ‘5kg’, adjust the scale when empty until it gives the correct weight of 5kg.

The plastic basin attached with four cords should be close to the floor for the child to feel safe. The use of the basin is comfortable for children and more hygienic because it can be easily washed.

The weight of the child (to the nearest 100 grams) is measured using the 25 kg Salter scale attached to the basin, following these steps:

- The scale must not be exposed to the sun;

For peer review only

- Readjust the scale to zero before each weighing;
- Explain to the mother that her child will be weighed naked in the basin to get her real weight and ask her to take off the child's clothes;
- The mother, helped by the CHW, places the child in the basin and remains next to the basin so that the child doesn't get too agitated and to reassure him or her;
- When the child does not move anymore, read the weight to the nearest 100 g (the needle should be at eye level) and note the weight in the register.

Height:

The height will be measured to the nearest 0.1 cm with a 130 cm wooden height board with graduated mm markings on each side. It will be measured in the prone position for children under 87 cm and in the standing position for children 87 cm or taller.

Children measuring less than 87 cm (prone position, see illustration below):

- Explain the procedure to the mother and ask her to remove the child's shoes and any ornaments in the child's hair.
- Place the board flat on the floor. The child lies down in the middle of the scale with the mother's help, with the child's feet on the side of the slider.
- The assistant holds the child's head between her/his hands at ear level and holds it firmly against the fixed part of the scale, the child's hair should be compressed. The child looks straight ahead.
- The measurer places his or her hands above the child's ankles, gently stretches the legs and places one hand on the child's thighs to prevent the child from bending his or her legs.
- Holding the legs firmly, he or she pushes the slider firmly flat against the soles of the child's feet.
- To read the measurement, the slider must be perpendicular to the axis of the scale and vertical. He then reads to the nearest 0.1cm and notes in the register.

Children measuring 87 cm or more (standing position) (see illustration below):

- Explain the procedure to the mother and ask her to remove the child's shoes and any ornaments in the hair;
- Place the standing scale on a solid flat surface and if possible against a wall.
- The child stands upright in the middle of the height board and touches the vertical plane of the board.
- The assistant holds the head, shoulders, buttocks, knees, ankles against the scale while the measurer positions the head and slide. He then reads to the nearest 0.1cm and notes in the register.

Calculating the weight for height Z-score

The weight for height Z-score (WHZ) should be recorded according to the weight for height Z-score table, which is from the national CMAM protocol (2016) and was used in the OptiMA trial. The nurse refers to the weight (in kg) and height (in cm) measured to read the child's WHZ.

How to use the weight/height ratio table?

Example: a child is 63 cm tall and weighs 6.5 kg.

For peer review only

- Take the table, look at the first column and search for the number 63cm (=height).
- Take a ruler or sheet of paper and place it under the number 63. On this line, find the weight corresponding to the child (in this case 6.5).
- Move up the corresponding column to determine which column it is. For this example, it corresponds to the column MEDIAN WEIGHT. For this example, the child's weight is normal in relation to his or her height. Therefore, the child's weight is appropriate for his or her height.

Example: a child is 78 cm tall and weighs 8.3 kg. This child is between column -2 and -3 Z-score or between MAM and SAM. He is too thin for his height; he is <-2 (less) and >-3 (more): he has MODERATE MALNUTRITION but NOT severe malnutrition. Record the WHZ as "<-2" in the register.

NOTE: It may happen that the height is not a round number, in which case rounding should be done according to the pattern below. For example: the size 80.1 and 80.2 are rounded to 80.0; 80.3, 80.4, 80.6 and 80.7 are rounded to 80.5; 80.8 and 80.9 are rounded to 81.0.

For peer review only

<u>The Mid Upper Arm Circumference (MUAC):</u>

- Ask the mother to remove the clothing that covers the child's left arm. Make a mark halfway between the shoulder and the left elbow (middle of the left arm). To do this, take a piece of string (or the MUAC itself), and place one end of the string on top of (arrow 1) and the other end on the elbow (arrow 2), making sure the string is taut. Then bend the string in half, bringing the end of the elbow back towards the end of the shoulder to the point halfway between the shoulder and the elbow.
- Another method can be used. Place the 0 of the MUAC (indicated by the 2 arrows) on top of the shoulder (arrow 4) and bring the other end towards the elbow (arrow 5). Read the number on the elbow to the nearest centimetre.
- Divide this number by two to get an estimate of the midpoint between the shoulder and the elbow. Make a mark on the arm with a pen at this value (arrow 6).
- Release the child's arm and place the MUAC around the arm at the mark. Make sure that the numbers are right side up. Make sure that the MUAC touches the skin (arrow 7).
- Check the tension on the MUAC. Make sure that the tension is correct, i.e. that the MUAC is not too tight (arm compressed) or too loose (the MUAC bracelet is not touching the skin all around the arm) (arrows 7 and 8).
- Repeat each step if necessary.
- When the MUAC is correctly positioned and the tension applied is good, read and pronounce the measurement out loud to the nearest 0.1cm (arrow 10).
- Record the measurement immediately.

<u>Checking for Nutritional Oedema:</u>

Nutritional oedema is detected in the following way:

- Normal pressure is exerted with the thumb on both feet for at least three seconds.
- If the thumb impression persists on both feet after removing the thumbs, then the child has oedema.
- Only children with bilateral oedema are recorded as having oedema.

You have to test with the pressure of your thumb! It is not enough just to look!

Severity of Oedema	Coding
**Mild Oedema**: present in both feet	**+**
**Moderate Oedema**: present in both feet and the lower part of both legs, and both hands and the lower part of both forearms. Intermediate between mild and severe oedema ++	**++**
**Severe Oedema:** generalized, including both feet, legs, hands, arms and face	**+++**

For peer review only

Additional file 2: RUTF ration per week according to Standard and OptiMA strategy

Standard RUTF ration per week		OptiMA RUTF ration per week			
	MUAC <115 or oedema or WHZ < -3 Z score		MUAC < 115 mm or oedema	MUAC [115-119 mm]	MUAC >=120 mm
**Weight (KG)**	**Number of RUTF sachet per week**	**Weight (KG)**	**Number of RUTF sachet per week**		
**3.0-3.4**	9	**3.0-3.4**	10	8	7
**3.5 - 4.9**	11	**3.5 - 4.0**	11	8	7
		**4.1 - 4.4**	12	9	7
		**4.5 - 4.9**	13	10	7
**5.0 - 6.9**	14	**5.0 - 5.4**	14	11	7
		**5.5 - 5.9**	15	12	7
		**6.0 - 6.4**	16	12	7
		**6.5 - 6.9**	17	13	7
**7.0 - 9.9**	21	**7.0 -7.4**	18	13	8
		**7.5 - 7.9**	19	14	8
		**8.0 - 8.4**	20	15	9
		**8.5 - 8.9**	22	15	9
		**9.0 - 9.4**	23	16	9
		**9.5 - 9.9**	24	17	9
**10.0 - 14.9**	28	**10.0 - 10.4**	25	18	10
		**10.5 - 10.9**	26	19	10
		**11.0 -11.4**	27	20	10
		**11.5 - 11.9**	29	21	10
		**12.0 - 12.4**	30	22	11
		**12.5 - 12.9**	31	22	11
		**13.0 - 13.4**	33	23	12
		**13.5 - 13.9**	34	24	12
		**14.0 - 14.4**	35	25	12
		**14.5 -14.9**	36	26	13
**15.0 - 19.9**	35	**15.0 - 15.4**	36	28	14
		**15.5 - 15.9**	36	28	14
		**16.0 - 16.4**	36	28	14
		**16.5 - 16.9**	36	28	14
		**17.0 - 17.4**	36	28	14
		**17.5 - 17.9**	36	28	14
		**18.0 - 18.4**	36	28	14
		**18.5 - 18.9**	36	28	14
		**19.0 - 19.4**	36	28	14
		**19.5 - 19.9**	36	28	14

For peer review only

SPIRIT Checklist for OptiMA-DRC protocol submission for publication

		Reporting Item	Page Number
**Administrative information**			
Title	#1	Descriptive title identifying the study design, population, interventions, and, if applicable, trial acronym	1
Trial registration	#2a	Trial identifier and registry name. If not yet registered, name of intended registry	2
Trial registration: data set	#2b	All items from the World Health Organization Trial Registration Data Set	2
Protocol version	#3	Date and version identifier	13
Funding	#4	Sources and types of financial, material, and other support	13
Roles and responsibilities: contributorship	#5a	Names, affiliations, and roles of protocol contributors	13
Roles and responsibilities: sponsor contact information	#5b	Name and contact information for the trial sponsor	1
Roles and responsibilities: sponsor and funder	#5c	Role of study sponsor and funders, if any, in study design; collection, management, analysis, and interpretation of data; writing of the report; and the decision to submit the report for publication, including whether they will have ultimate authority over any of these activities	11
Roles and responsibilities: committees	#5d	Composition, roles, and responsibilities of the coordinating centre, steering committee, endpoint adjudication committee, data management team, and other individuals or groups overseeing the trial, if applicable (see Item 21a for data monitoring committee)	11, 14
**Introduction**			
Background and rationale	#6a	Description of research question and justification for undertaking the trial, including summary of relevant studies (published and unpublished) examining benefits and harms for each intervention	3,4,5
Background and rationale: choice of comparators	#6b	Explanation for choice of comparators	4
Objectives	#7	Specific objectives or hypotheses	5

For peer review only

Trial design	#8	Description of trial design including type of trial (eg, parallel group, crossover, factorial, single group), allocation ratio, and framework (eg, superiority, equivalence, non-inferiority, exploratory)	5
**Methods: Participants, interventions, and outcomes**			
Study setting	#9	Description of study settings (eg, community clinic, academic hospital) and list of countries where data will be collected. Reference to where list of study sites can be obtained	6
Eligibility criteria	#10	Inclusion and exclusion criteria for participants. If applicable, eligibility criteria for study centres and individuals who will perform the interventions (eg, surgeons, psychotherapists)	6,7
Interventions: description	#11a	Interventions for each group with sufficient detail to allow replication, including how and when they will be administered	9,10
Interventions: modifications	#11b	Criteria for discontinuing or modifying allocated interventions for a given trial participant (eg, drug dose change in response to harms, participant request, or improving / worsening disease)	8,11
Interventions: adherance	#11c	Strategies to improve adherence to intervention protocols, and any procedures for monitoring adherence (eg, drug tablet return; laboratory tests)	10
Interventions: concomitant care	#11d	Relevant concomitant care and interventions that are permitted or prohibited during the trial	9
Outcomes	#12	Primary, secondary, and other outcomes, including the specific measurement variable (eg, systolic blood pressure), analysis metric (eg, change from baseline, final value, time to event), method of aggregation (eg, median, proportion), and time point for each outcome. Explanation of the clinical relevance of chosen efficacy and harm outcomes is strongly recommended	7,19,20
Participant timeline	#13	Time schedule of enrolment, interventions (including any run-ins and washouts), assessments, and visits for participants. A schematic diagram is highly recommended (see Figure)	12,21 (figure 1)
Sample size	#14	Estimated number of participants needed to achieve study objectives and how it was determined, including clinical and statistical assumptions supporting any sample size calculations	7
Recruitment	#15	Strategies for achieving adequate participant enrolment to reach target sample size	7,8
**Methods: Assignment of interventions (for controlled trials)**			

For peer review only

Allocation: sequence generation	#16a	Method of generating the allocation sequence (eg, computer-generated random numbers), and list of any factors for stratification. To reduce predictability of a random sequence, details of any planned restriction (eg, blocking) should be provided in a separate document that is unavailable to those who enrol participants or assign interventions	8
Allocation concealment mechanism	#16b	Mechanism of implementing the allocation sequence (eg, central telephone; sequentially numbered, opaque, sealed envelopes), describing any steps to conceal the sequence until interventions are assigned	8
Allocation: implementation	#16c	Who will generate the allocation sequence, who will enrol participants, and who will assign participants to interventions	8
Blinding (masking)	#17a	Who will be blinded after assignment to interventions (eg, trial participants, care providers, outcome assessors, data analysts), and how	10
Blinding (masking): emergency unblinding	#17b	If blinded, circumstances under which unblinding is permissible, and procedure for revealing a participant's allocated intervention during the trial	"n/a" no blinding
**Methods: Data collection, management, and analysis**			
Data collection plan	#18a	Plans for assessment and collection of outcome, baseline, and other trial data, including any related processes to promote data quality (eg, duplicate measurements, training of assessors) and a description of study instruments (eg, questionnaires, laboratory tests) along with their reliability and validity, if known. Reference to where data collection forms can be found, if not in the protocol	10
Data collection plan: retention	#18b	Plans to promote participant retention and complete follow-up, including list of any outcome data to be collected for participants who discontinue or deviate from intervention protocols	10
Data management	#19	Plans for data entry, coding, security, and storage, including any related processes to promote data quality (eg, double data entry; range checks for data values). Reference to where details of data management procedures can be found, if not in the protocol	8,10
Statistics: outcomes	#20a	Statistical methods for analysing primary and secondary outcomes. Reference to where other details of the statistical analysis plan can be found, if not in the protocol	11
Statistics: additional analyses	#20b	Methods for any additional analyses (eg, subgroup and adjusted analyses)	11

For peer review only

Statistics: analysis population and missing data	#20c	Definition of analysis population relating to protocol non-adherence (eg, as randomised analysis), and any statistical methods to handle missing data (eg, multiple imputation)	11
**Methods: Monitoring**			
Data monitoring: formal committee	#21a	Composition of data monitoring committee (DMC); summary of its role and reporting structure; statement of whether it is independent from the sponsor and competing interests; and reference to where further details about its charter can be found, if not in the protocol. Alternatively, an explanation of why a DMC is not needed	10
Data monitoring: interim analysis	#21b	Description of any interim analyses and stopping guidelines, including who will have access to these interim results and make the final decision to terminate the trial	11
Harms	#22	Plans for collecting, assessing, reporting, and managing solicited and spontaneously reported adverse events and other unintended effects of trial interventions or trial conduct	10,11
Auditing	#23	Frequency and procedures for auditing trial conduct, if any, and whether the process will be independent from investigators and the sponsor	11,13 (national ethic committee audit)
**Ethics and dissemination**			
Research ethics approval	#24	Plans for seeking research ethics committee / institutional review board (REC / IRB) approval	11,13
Protocol amendments	#25	Plans for communicating important protocol modifications (eg, changes to eligibility criteria, outcomes, analyses) to relevant parties (eg, investigators, REC / IRBs, trial participants, trial registries, journals, regulators)	13
Consent or assent	#26a	Who will obtain informed consent or assent from potential trial participants or authorised surrogates, and how (see Item 32)	11,12
Consent or assent: ancillary studies	#26b	Additional consent provisions for collection and use of participant data and biological specimens in ancillary studies, if applicable	"n/a", no ancillary study planned
Confidentiality	#27	How personal information about potential and enrolled participants will be collected, shared, and maintained in order to protect confidentiality before, during, and after the trial	11
Declaration of interests	#28	Financial and other competing interests for principal investigators for the overall trial and each study site	13
Data access	#29	Statement of who will have access to the final trial dataset, and disclosure of contractual agreements that limit such access for investigators	13

For peer review only

Ancillary and post trial care	#30	Provisions, if any, for ancillary and post-trial care, and for compensation to those who suffer harm from trial participation	"n/a" participants are covered by insurance of the trial
Dissemination policy: trial results	#31a	Plans for investigators and sponsor to communicate trial results to participants, healthcare professionals, the public, and other relevant groups (eg, via publication, reporting in results databases, or other data sharing arrangements), including any publication restrictions	11
Dissemination policy: authorship	#31b	Authorship eligibility guidelines and any intended use of professional writers	13, no use of professional writers planned
Dissemination policy: reproducible research	#31c	Plans, if any, for granting public access to the full protocol, participant-level dataset, and statistical code	13
**Appendices**			
Informed consent materials	#32	Model consent form and other related documentation given to participants and authorised surrogates	13
Biological specimens	#33	Plans for collection, laboratory evaluation, and storage of biological specimens for genetic or molecular analysis in the current trial and for future use in ancillary studies, if applicable	"n/a" no biological specimens collected

For peer review only
